# Diversity and future perspectives of Mediterranean deep-water oyster reefs

**DOI:** 10.1038/s41598-024-77641-x

**Published:** 2024-12-28

**Authors:** Giorgio Castellan, Lorenzo Angeletti, Marco Taviani

**Affiliations:** 1 Institute of Marine Sciences, National Research Council (CNR-ISMAR), Bologna, Italy; 2 Institute for Marine Biological Resources and Biotechnology, National Research Council (CNR- IRBIM), Ancona, Italy; 3 NBFC - National Biodiversity Future Centre, Palermo, Italy; 4 Stazione Zoologica Anton Dohrn, Naples, Italy

**Keywords:** Reefs, Oyster reefs, Biodiversity, Functional diversity, Mediterranean Sea, Mesophotic zone, Marine biology, Climate change, Ocean sciences, Biodiversity, Community ecology

## Abstract

Anthropogenic and climate factors are increasingly affecting the composition and functions of many marine biogenic reefs globally, leading to a decline in associated biodiversity and ecosystem services. Once dominant ecological component, modern oyster reefs in the Mediterranean and Black Sea and the Atlantic Ocean have already been profoundly altered by overharvesting, habitat loss and the introduction of alien species. Far less known are deep-water oyster reefs, which can however form substantial biogenic structures below 30 m depth. Here we analyze the diversity of benthic assemblages associated with deep-water oyster reefs formed by the gryphaeid *Neopycnodonte cochlear*, and other mesophotic habitats in the central Mediterranean Sea using a taxonomic and functional approach. Our findings suggest that deep-water oyster reefs may act as hotspots of biodiversity and ecological functions in the Mediterranean Sea under current conditions, having also an edge in survival in a changing ocean.

## Introduction

The decline in the extent, structure, and functioning of the world’s marine ecosystems is arguably among the largest ecological crises of our time. Numerous lines of evidence prove that degradation, such as biodiversity loss, population collapse, and invasion of exotic species is now apparent in most ecosystems^1^. Causes include the increase in nutrient loads and zones of low dissolved oxygen^2^, pollution^3^, fisheries exploitation^4,5^, physical habitat destruction^6,7^, ocean acidification^8–10^, diseases^11^, and warming^12^. These anthropogenic and climate pressures may result in the occurrence of new abiotic conditions that can lead to changes in species composition and relative abundances, and to the ecological success of non-native over indigenous species^13^. When these new ecological configurations persist across time, altered ecosystems are unlikely to return to historic conditions, leading to the establishment of “novel ecosystems”^14^.

A representative case is marine biogenic reefs, highly productive and diverse three-dimensional environments formed by autogenic ecosystem engineers (*sensu* Jones et al.^15^), which are currently undergoing major changes on the global scale^16^.

Around one-third of the world’s reefs built by tropical corals has been severely degraded by anthropogenic and climate factors^17^. Increasing seawater temperature induces corals to expel the pigmented microalgal endosymbionts (zooxanthellae) from which they derive much of their nutrition, causing corals to become pale or white and ultimately leading to death if thermal stress is severe and prolonged^17^. Additionally, the reduction in the saturation state of aragonite, the form of calcium carbonate used by coral to build their skeleton, due to ocean acidification poses a further major threat, since reducing the ability of corals to form their skeletons^18^.

Such changes are influencing the appearance and functional ecology of coral reefs, driving the establishment of new ecological configurations that render unlikely a return to pristine status^19–22^. Whilst some reefs will continue to be dominated by calcifying organisms but characterized by a different set of species and functions^23^, other reef ecosystems are already experiencing regime shifts towards a different ecological state^24^.

In temperate situations, oysters, individuals in the families Ostreidae and Gryphaeidae, represented in the past one of the dominant structural and ecological components, carpeting considerable areas of the seafloor in coastal areas during the Mesozoic and Cenozoic^25–28^, and in deeper waters, since the Middle Miocene^29–31^. Nowadays, in the Mediterranean and Black Sea and Atlantic Ocean, the distribution and extension of oyster reefs have been heavily altered by human activities^32^.

Despite heat waves may have major consequences^33^, the absence of symbiotic relations with zooxanthellae and the calcitic structure of the skeleton seem to confer to oysters a higher tolerance to ocean warming and acidification than corals^34^. Causes of the decline of autochthonous shallow-water oyster reefs must be instead likely sought in commercial overharvesting and the imposition of the alien Pacific oyster *Magallana gigas* (Thunberg 1793)^35^ (formerly *Crassostrea gigas*), which has led to an ecological reconfiguration of indigenous reefs.

Deeper in the water column, oyster reefs in mesophotic and bathyal situations are formed by members of the Family Gryphaeidae, mainly *Neopycnodonte cochlear* (Poli, 1795) and *Neopycnodonte zibrowii* Gofas Salas and Taviani, 2009, respectively. Literature records indicate that deep-water oyster reefs (DWOR) can build large three-dimensional structures providing habitats for various species^36–49^. Located below 30 m depth, DWOR seem to be less exposed to climatic stressors that affect shallow-water environments (such as heatwaves and warming), and actually the major threats to these reefs appear to be mainly related to fishing activities^36^.

A potential edge of DWOR for future survival in a changing ocean along with the scarcity of information render crucial to broadening the understanding of the diversity of deep-water oyster reef ecosystems and the ecological functions they provide.

Here we analyze the diversity of benthic assemblages associated with DWOR formed by *N. cochlear*, and compare them with other benthic assemblages located in the central Mediterranean Sea, using both taxonomic and functional approach. Our comparison aims at investigating whether these overlooked reefs serve as hotspots of biodiversity and ecological functions in the Mediterranean Sea. We also anticipate the potential fate of DWOR in the future ocean.

## Results

### Habitat characterization and taxonomic composition

**Fig. 1:**
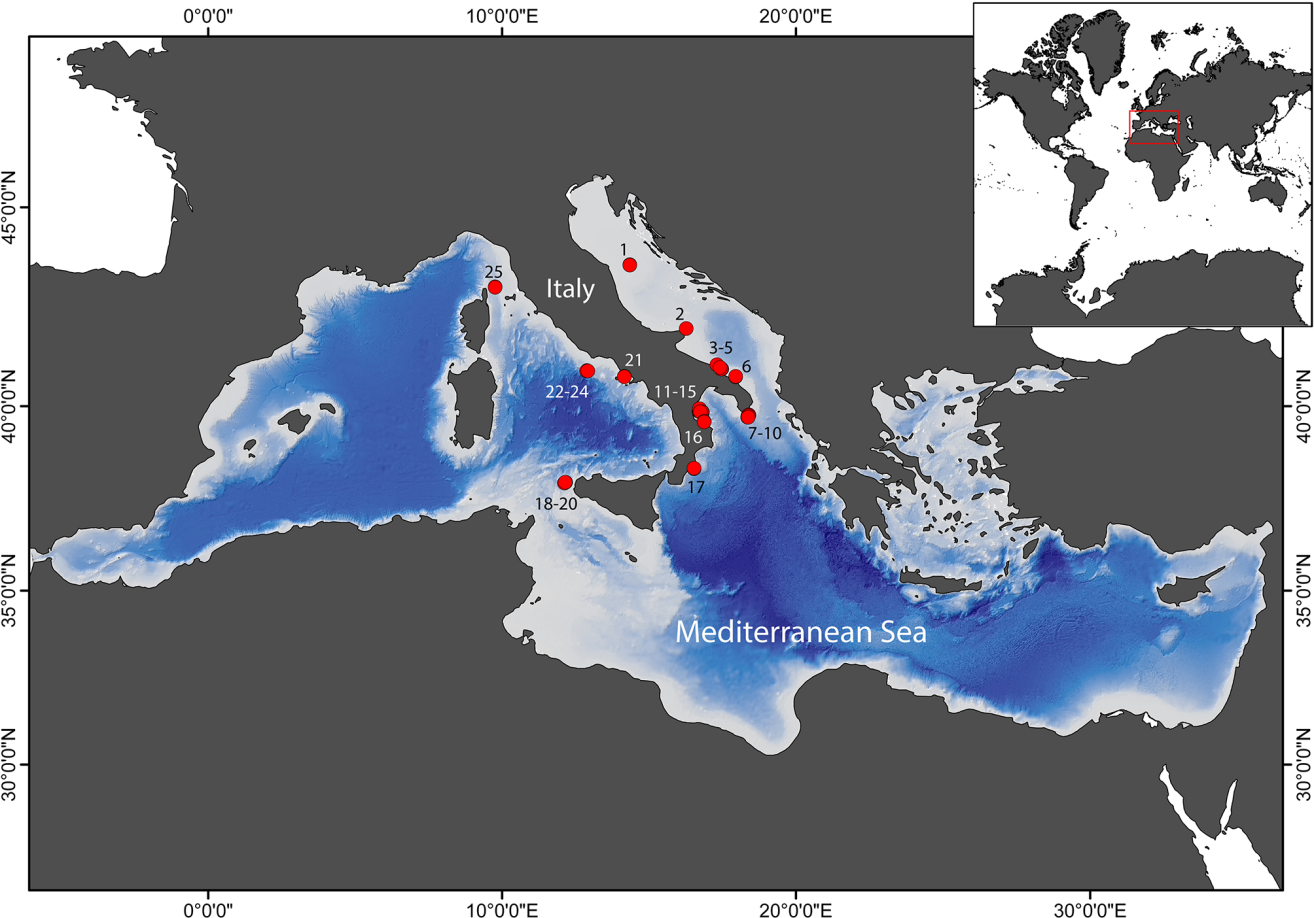
Location of the 25 ROV dives performed at mesophotic depth in the central Mediterranean Sea. Map created using ArcGIS 10.8 (ESRI^®^www.esri.com).

**Fig. 2:**
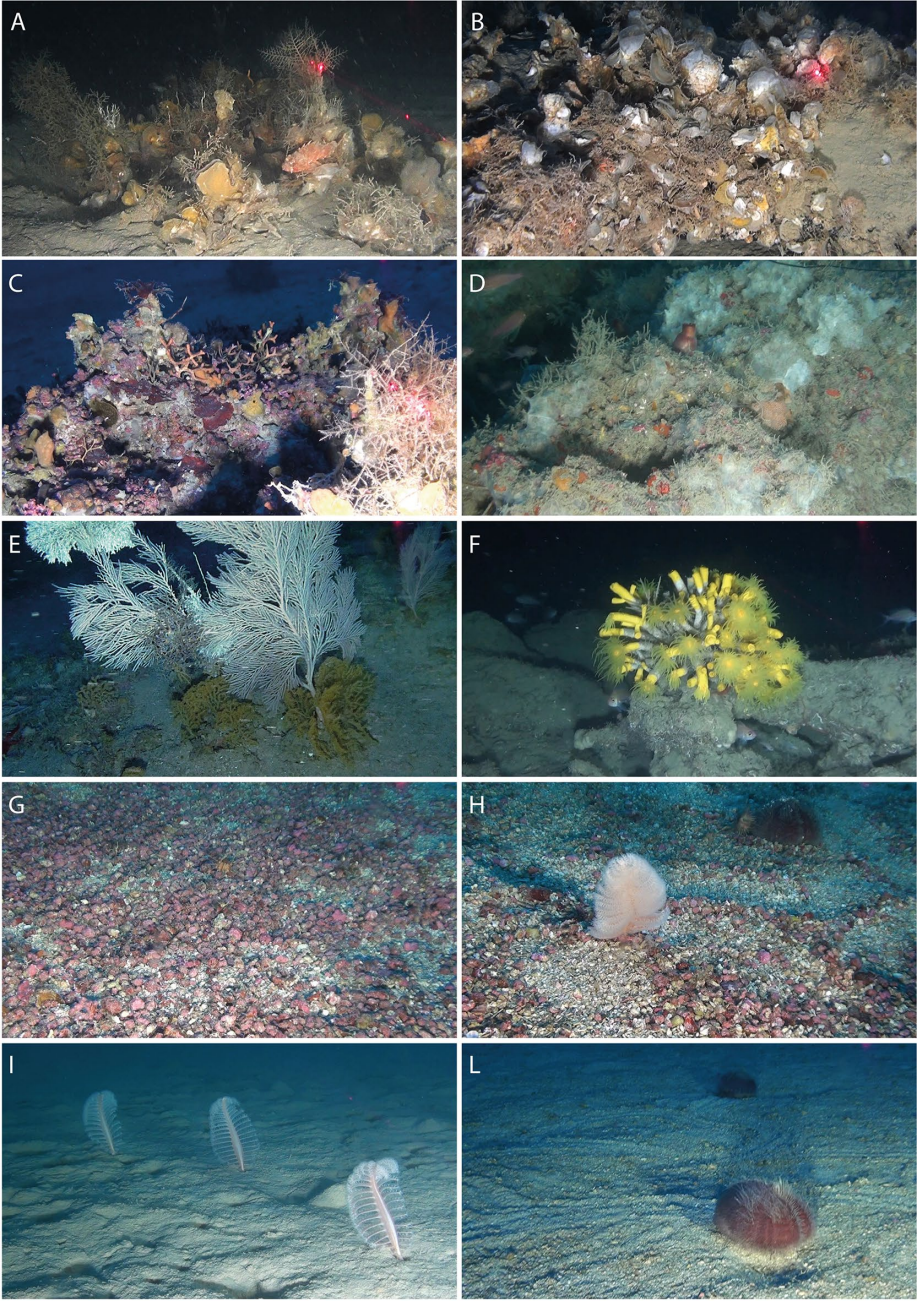
Examples of the benthic assemblages considered in this study. Deep-water oyster reefs (DWOR) in the Adriatic Sea at ca. 90 m (**A**, ID 1), and at ca. 100 m (**B**, ID 5); coralligenous formations in the Ionian Sea at ca. 90 m (**C**, ID 11) and at ca. 70 m (**D**, ID 17); cnidarian-dominated assemblages at ca. 110 m in the Tyrrhenian Sea (**E**, ID 18) and at ca. 150 m in the Ionian Sea (**F**, ID 14); rhodolith beds at ca. 70 m in the Tyrrhenian Sea (**G**,**H**, ID 19); soft-bottom assemblages in the Tyrrhenian Sea at ca. 60 m (**I**, ID 16) and at ca. 100 m (**L**, ID 25). Numbers refer to IDs in (Table 1).

Mesophotic habitats in 25 locations in the central Mediterranean Sea were surveyed using Remotely Operated Vehicles (ROV, Fig. 1). Five different benthic assemblages were identified (Fig. 2), classified based on principal/dominant taxonomic component: deep-water oyster reefs assemblages (DWOR); coralligenous assemblages (C); cnidarian-dominated assemblages (CN); rhodolith bed assemblages (RB); and soft-bottom assemblages (SB). Collected videos recorded more than 27 km of seafloor, resulting in more than 12,500 frames extracted (Table 1). The 76.7% of frames imaged portions of the seafloor dominated by soft bottoms, 53.7% of which constituted by mud and sands while 23% hosted rhodolith beds. The 23.3% of remaining extracted images showed seafloor characterized by hard substrate, either in form of continuous hard bottom or blocks elevating from the seabed. Of these, 3.2% and 4.7% hosted coralligenous formations and deep-water oyster reefs (DWOR), respectively. The analysis of frames extracted from video recordings for taxonomic identification registered a total of 17,263 megabenthic organisms belonging to 291 different taxa. The 78.3% of the taxa were classified at the species level (49.8%) or genus level (28.5%), while the remaining were identified as morphospecies and classified with higher taxonomic levels: family (6.5%), order (2.7%), and class (12.3%). The complete list of identified taxa is provided in Tab. S1. Porifera and Cnidaria represented the major contributors to the biodiversity of the explored sites, corresponding to 30.9% and 15.1% of taxa identified, respectively. Echinodermata were frequently observed (10.3%), together with Mollusca (8.6%), Crustacea (6.5%), Ascidiacea (5.2%), and Bryozoa (4.8%). Also, Annelida colonizing the seafloor and epibionts on other sessile fauna were abundant (4.5%). Although not included for further analysis, the nektonic fauna was well represented, with 34 taxa identified. In terms of individuals or colonies, Porifera were the most abundant group (38.2%), followed by Cnidaria (24.4%), Bryozoa (14.9%), and Echinodermata (12.3%). The remaining groups counted approximately 8% of the total number of organisms.

**Table 1:** Technical information of the 25 ROV videos performed on mesophotic assemblages. Table reports the assemblage category and number of frames extracted.

ID	ROV	Location	Area	Date	Lat	Long	Lenght (m)	Depth range (m)	Assemblage	Extr. Frames
1	MS17_III_110	Bonaccia field	Adriatic Sea	13/08/2017	41° 59’ 37.24"N	16° 15’ 8.66"E	862.49	87–90	DWOR	440
2	MS15_47	Off Vieste	Adriatic Sea	10/11/2015	40° 57’ 44.56"N	17° 27’ 34.57"E	1127.32	50–60	DWOR	296
3	MS17_III_115	Off Monopoli	Adriatic Sea	15/08/2017	39° 45’ 50.71"N	18° 23’ 25.83"E	984.76	100–103	DWOR	449
4	MS15_79	Off Monopoli	Adriatic Sea	12/11/2015	39° 45’ 16.39"N	18° 21’ 59.03"E	994.92	72–80	C	614
5	MS17_II_180	Off Monopoli	Adriatic Sea	05/08/2017	39° 35’ 11.73"N	16° 52’ 6.84"E	1564.42	92–110	DWOR	644
6	MS17_II_165	Off Brindisi	Adriatic Sea	04/08/2017	38° 20’ 37.82"N	16° 31’ 13.99"E	1359.06	102–105	SB	737
7	MS15_127	Off Santa Maria di Leuca	Ionian Sea	16/11/2015	40° 54’ 41.63"N	12° 52’ 59.28"E	1230.8	70–95	DWOR	727
8	MS15_118	Off Santa Maria di Leuca	Ionian Sea	15/11/2015	40° 54’ 30.55"N	12° 52’ 6.37"E	1129.15	90–98	DWOR	455
9	MS17_II_117	Off Santa Maria di Leuca	Ionian Sea	31/07/2017	37° 58’ 31.29"N	12° 8’ 24.23"E	1179.23	124–138	DWOR	599
10	MS17_II_115	Off Santa Maria di Leuca	Ionian Sea	31/07/2017	37° 56’ 59.78"N	12° 7’ 15.34"E	896.67	108–126	DWOR	507
11	MS17_II_83	Amendolara Seamount	Ionian Sea	28/07/2017	37° 58’ 27.72"N	12° 8’ 53.24"E	1424.7	91–97	SB	561
12	MS16_II_89	Amendolara Seamount	Ionian Sea	10/09/2016	40° 54’ 46.31"N	12° 54’ 29.29"E	1151.07	67–88	C	680
13	MS16_II_83	Amendolara Seamount	Ionian Sea	10/09/2016	39° 51’ 32.46"N	16° 41’ 59.61"E	647.65	67–83	C	653
14	MS17_II_93	Amendolara Seamount	Ionian Sea	29/07/2017	40° 45’ 59.2"N	14° 9’ 24.18"E	1370.36	132–168	CN	669
15	MS17_II_92	Amendolara Seamount	Ionian Sea	29/07/2017	43° 2’ 19.91"N	9° 45’ 1.81"E	859.29	170–190	CN	489
16	MS15_144	Off Crotone	Ionian Sea	18/11/2015	39° 55’ 57.17"N	16° 42’ 42.99"E	855.98	60–65	SB	905
17	MS15_184	Off Rocella Ionica	Ionian Sea	20/11/2015	39° 49’ 48.75"N	16° 48’ 7.5"E	986.38	68–79	C	570
18	MS16_186	Egadi Islands	Tyrrhenian Sea	01/08/2016	39° 50’ 49.94"N	16° 47’ 47.87"E	840.76	104–118	CN	705
19	MS16_197	Egadi Islands	Tyrrhenian Sea	02/08/2016	39° 44’ 0.87"N	18° 22’ 15.13"E	1086.01	70–75	RB	550
20	MS16_203	Egadi Islands	Tyrrhenian Sea	03/08/2016	39° 42’ 19.07"N	18° 21’ 19.25"E	1448.76	95–115	CN	877
21	MS17_I_103	Gulf of Naples	Tyrrhenian Sea	13/07/2017	40° 45’ 47.31"N	17° 56’ 32.11"E	1522.48	113–119	SB	842
22	MS16_21	Pontine Islands	Tyrrhenian Sea	18/07/2016	41° 0’ 11.31"N	17° 24’ 15.93"E	670.68	45–68	RB	520
23	MS16_128	Pontine Islands	Tyrrhenian Sea	27/07/2016	43° 35’ 29.4"N	14° 20’ 7.91"E	827.24	65–72	RB	678
24	MS16_142	Pontine Islands	Tyrrhenian Sea	27/07/2016	41° 4’ 9.69"N	17° 18’ 9.76"E	1240.14	55–80	RB	638
25	MS17_I_136	Off Capraia	Tyrrhenian Sea	17/07/2017	39° 50’ 38.28"N	16° 43’ 49.02"E	1376.04	98–105	SB	876

* C* coralligenous assemblages, * SB* soft-bottom assemblages, * CN* cnidarian-dominated assemblages, * DWOR* deep-water oyster-reef assemblages, rhodolith-bed assemblages.

The ANOSIM analyses provided evidence that the assemblages were significantly different in terms of taxonomic composition but showing some overlaps among groups (*p* < 0.01, *R* = 0.38). This overlap appeared to reduce when considering only sites within depth range 55–100 m (*p* < 0.01, *R* = 0.44), and sites within 100–200 m (*p* < 0.01, *R* = 0.6).

**Fig. 3:**
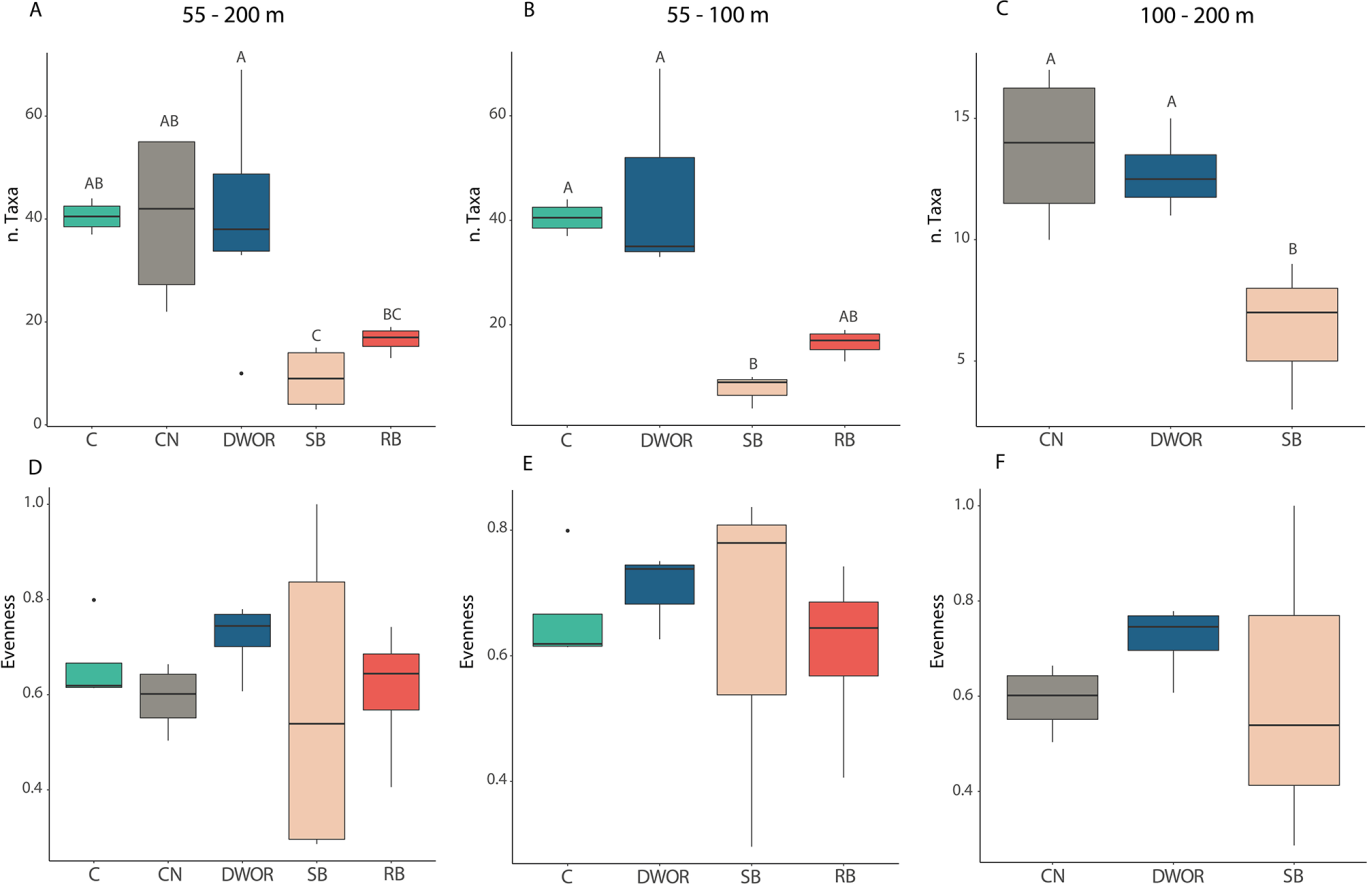
Boxplot of taxonomic richness and evenness considering all dives, dives within the depth range 55–100 m, and dives within the depth range 100–200 m. Boxes extend from the 25th to the 75th percentile with the horizontal line representing median value. The vertical lines indicate the most extreme values within 1.5 interquartile range of the 25 and 75th percentile. Letters refer to significant differences.

Coralligenous (C) and cnidarian (CN) assemblages presented the highest taxonomic richness (Fig. 3), with 24.15 ± 4.6 and 21.16 ± 4.2 taxa identified with 100 frames (Table 2). The DWOR reported slightly lower values, counting on average 18.40 ± 2.6 taxa with the same number of samples. No significant difference in the taxonomic richness among C, CN and DWOR was observed. The richness of taxa associated with rodolith-bed (RB) and soft-bottom assemblages (SB) was strongly lower, showing values of 6.83 ± 2.5 and 5.2 ± 0.2, respectively. The difference in richness of taxa among assemblages was significant (ANOVA test, *p* < 0.01). The Tukey post hoc test showed that these differences were significant between CN, C, DWOR and SB, and between DWOR and RB. No significant differences in Pielou’s evenness among assemblages were observed.

**Table 2:** Taxonomic and functional richness and standard errors with 100 frames resulting from species accumulation curves for ROV dives used in this study.

ROV	Assemblage	Taxa Rich	Taxa Rich / Ass	Pielou’s Evenness	Func Rich	Func Rich / Ass	Func Evenness
MS15_79	C	16.82 ± 4.2	22.32 ± 3.72	0.80	8.91 ± 1.7	9.09 ± 0.9	0.30
MS15_184	C	17.25 ± 3.4		0.62	7.68 ± 1.7		0.23
MS16_II_83	C	32.83 ± 2.1		0.61	11.68 ± 0.5		0.29
MS16_II_89	C	22.38 ± 3.1		0.62	8.09 ± 0.84		0.25
MS16_186	CN	30.33 ± 3.6	21.16 ± 4.18	0.57	11.21 ± 1.4	10.12 ± 0.59	0.18
MS16_203	CN	26.12 ± 3.9		0.64	10.83 ± 1.6		0.23
MS17_II_92	CN	13.39 ± 1.6		0.50	8.58 ± 1.3		0.23
MS17_II_93	CN	14.81 ± 1.8		0.66	9.87 ± 0.9		0.22
MS15_47	DWOR	5.13 ± 1.9	18.06 ± 2.33	0.78	2.62 ± 0.6	8.22 ± 0.95	0.25
MS15_118	DWOR	16.1 ± 3.2		0.74	7.98 ± 1.4		0.30
MS15_127	DWOR	25.5 ± 4.4		0.63	10.63 ± 1.3		0.22
MS17_II_115	DWOR	25.64 ± 2.8		0.61	11.64 ± 1.4		0.26
MS17_II_117	DWOR	18.81 ± 3.7		0.78	7.21 ± 1.2		0.21
MS17_II_180	DWOR	15.66 ± 2.5		0.73	8.10 ± 1.1		0.22
MS17_III_110	DWOR	16.32 ± 3.2		0.75	8.50 ± 1.3		0.27
MS17_III_115	DWOR	21.33 ± 3.2		0.77	9.07 ± 1.3		0.22
MS15_144	SB	1.45 ± 0.5	3.07 ± 1.04	0.30	2.84 ± 0.8	3.14 ± 0.49	0.06
MS17_I_103	SB	3.7 ± 1.3		0.53	4.22 ± 1.3		0.42
MS17_I_136	SB	6.48 ± 1.4		0.29	3.69 ± 0.5		0.32
MS17_II_165	SB	0.41 ± 0.6		0.98	1.40 ± 0.6		0.83
MS17_II_83	SB	3.32 ± 1.1		0.84	3.54 ± 1.3		0.64
MS16_21	RB	5.61 ± 1.6	5.33 ± 0.24	0.67	5.57 ± 1.0	4.32 ± 0.58	0.35
MS16_128	RB	5.65 ± 1.9		0.74	4.25 ± 1.1		0.43
MS16_197	RB	5.43 ± 1.4		0.41	2.80 ± 0.8		0.42
MS16_142	RB	4.64 ± 2.5		0.62	4.64 ± 1.6		0.32

**Fig. 4:**
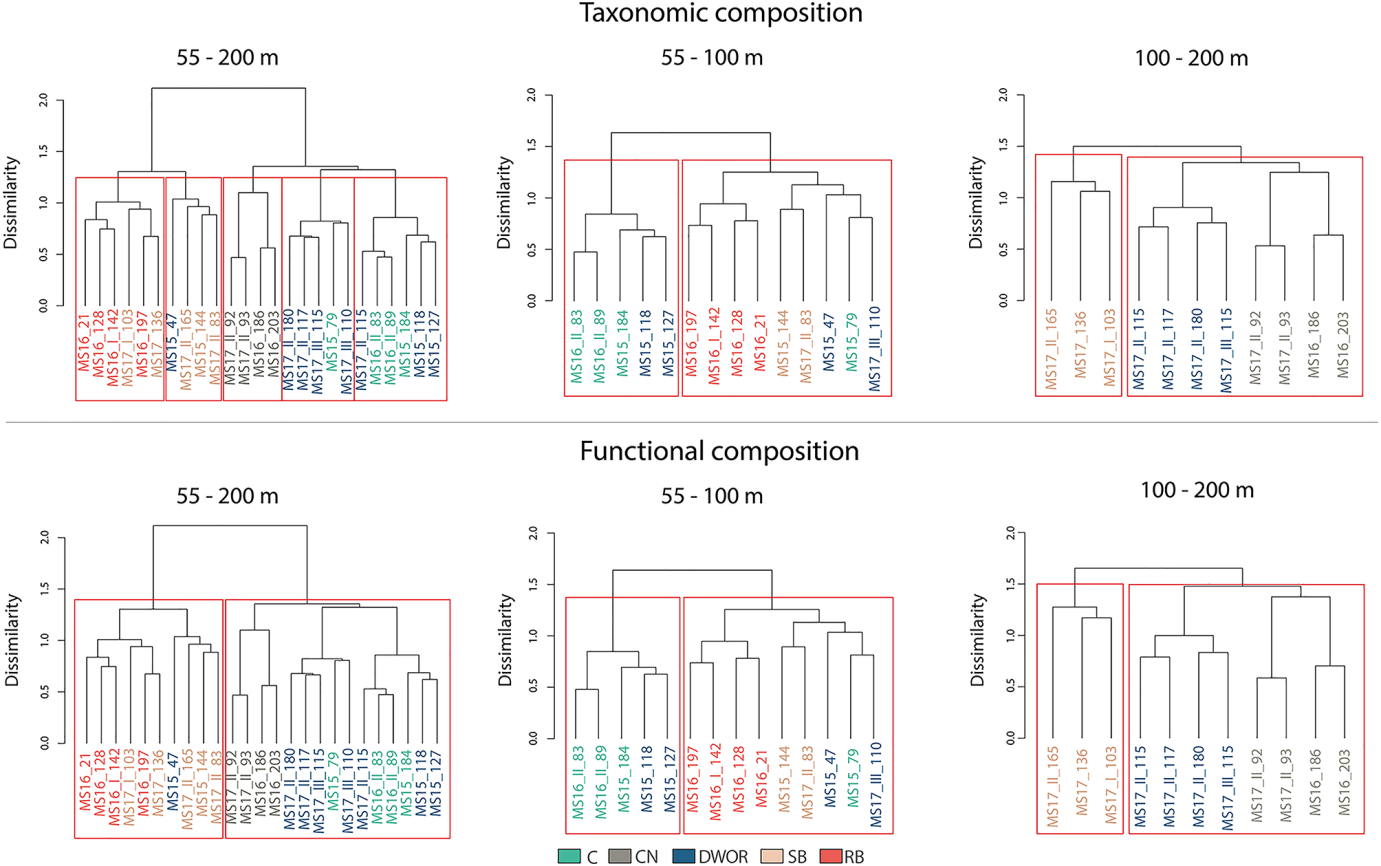
Dendrograms showing Ward’s clustering constructed over Bray-Curtis dissimilarity of square-root transformed taxonomic (up) and functional (bottom) abundance data considering all dives, dives within the depth range 55–100 m, and dives within the depth range 100–200 m. Red squares delineate groups identified with silhouette function.

The hierarchical clustering exploring site similarity identified five major groups, grouping sites in line with the classification made through video analysis but not completely discriminating between RB and SB, and between DWOR and C, which appeared to mingle (Fig. 4).

Considering assemblages in the depth range 55–100 m, the taxonomic richness of C and DWOR assemblages was significantly different from that of SB (Fig. 3). No significant differences were observed in Pielou’s evenness among assemblages. Two main clusters were again identified by the hierarchical clustering, the first including only C and DWOR assemblages and the second largely composed by SB and RB but including also a C and two DWOR assemblages.

The taxonomic richness of CN and DWOR assemblages was significantly higher with respect to SB in the depth range 100–200 m, and the hierarchical clustering separated CN and DWOR from SB (Fig. 4). Pielou’s evenness was not significantly different among assemblages.

### Functional composition

The analysis of functional diversity associated with explored assemblages resulted in 22 different functional entities (FE, Tab. S2). The classification of taxa depending on their functional characteristics registered (i) two categories of adult body dimension (macro- and megafauna), (ii) four adult motility categories (sessile, facultatively motile, vagile, and swimmers), (iii) six different strategies of feeding (deposit feeders, grazers, filter feeders, suspension feeders, scavenger/predators, and photosynthetic organisms), (iv) two types of adult habits (benthic and pelagic), (v) three categories of organism aggregation (single, colonial and gregarious), either (vi) capable to build habitat or not.

**Fig. 5:**
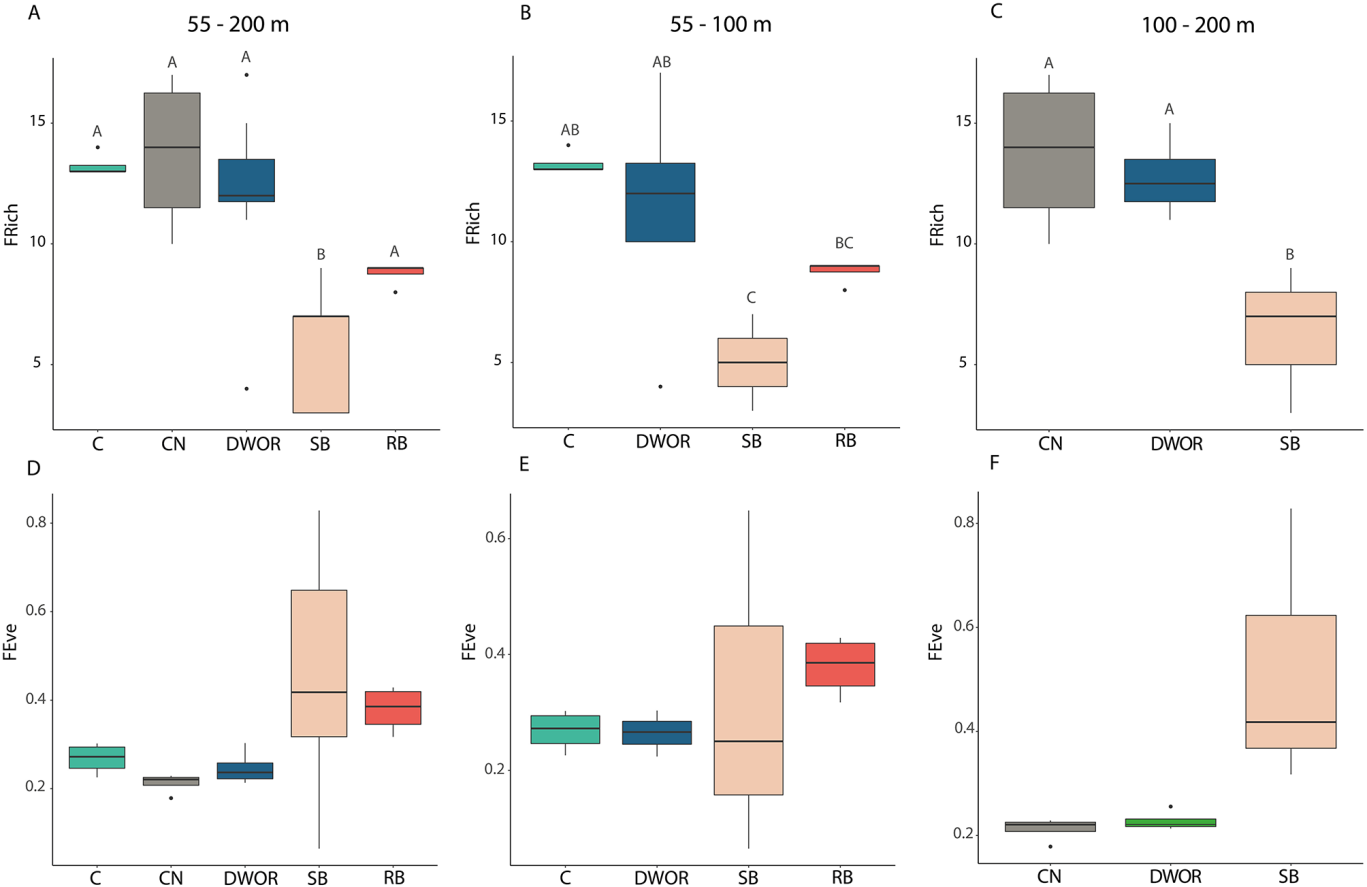
Boxplot of functional richness and evenness considering all dives, dives within the depth range 55–100 m, and dives within the depth range 100–200 m. Boxes extend from the 25th to the 75th percentile with the horizontal line representing median value. The vertical lines indicate the most extreme values within 1.5 interquartile range of the 25th and 75th percentile. Letters refer to significant differences.

The ANOSIM analysis reported significant differences among assemblages but with overlaps in terms of functional composition when considering the dives together (*p* < 0.01, *R* = 0.44) and by depth range (55–100 m: *p* < 0.01, *R* = 0.46; 100–200 m: *p* < 0.01, *R* = 0.46). The CN assemblages reported the highest functional richness (Frich), with an average of 10.12 ± 0.59 FEs (Fig. 5). Similar richness values were observed for C assemblages, which presented 9.09 ± 0.9 FEs on average. A slightly lower Frich was detected for DWOR assemblages, hosting an average of 8.22 ± 0.95 FEs. The RB and SB assemblages presented the lowest Frich values and were characterized by 4.32 ± 0.58 and 3.14 ± 0.49 FEs, respectively.

The Kruskal-Wallis and post hoc Dunn tests revealed significant differences in the functional richness between C, CN, DWOR, RB and SB. The hierarchical clustering exploring the similarity between sites in terms of functional composition detected two major clusters (Fig. 4): C, CN, and DWOR composed the first cluster, while the second grouped RB and SB assemblages, except one DWOR assemblage.

The SIMPER analysis reported that differences in functional composition among the two groups were mainly explained by variations in the abundance of non-habitat building solitary filter feeders and habitat building suspension feeders both solitary and colonial (Tab. S3).

Within the depth range 55–100 m, C and DWOR showed a significantly higher functional richness compared to SB assemblages. In terms of functional composition, the hierarchical clustering segregated DWOR and C separately from SB and RB assemblages, except for two assemblages (Fig. 5). These differences were mainly related with variation in the abundance of megafaunal suspension feeders both colonial and solitary, and both habitat and non-habitat building (Tab. S3).

Both functional richness and composition of CN and DWOR assemblages significantly differed from those of SB assemblages in the depth range 100–200 m (Figs. 4 and 5).

The SIMPER reported different abundances of megafaunal solitary filter feeders non-habitat builders, megafaunal benthic scavengers/predators and megafaunal colonial suspension feeders non-habitat builders (Tab. S3).

## Discussion

Shallow-water oysters are known to form dense assemblages and create biogenic habitats that enhance the structural complexity of the seafloor and represent habitat for many organisms^50^.

In line with previous findings, our analysis of the taxonomic diversity provides evidence that also deep-water reefs formed by *N. cochlear* can harbor highly biodiverse assemblages^35–39^. Species richness of deep-water oyster reefs (DWOR) assemblages was high and comparable to that of coralligenous (C), and cnidarian-dominated (CN) assemblages, widely recognized as hotspots of biodiversity at mesophotic depths in the Mediterranean Sea^51,52^.

Consistently, the hierarchical clustering based on the taxonomic composition was not completely able to distinguish DWOR from C and CN assemblages, which formed mixed groups. Dive MS15_47 represented an exception since, despite being performed DWOR assemblage, was grouped with SB and RB. In this dive, the ROV surveyed the target assemblages only partially, with just a few frames imaging DWOR situations whilst most of the recordings showed mobile bottoms. Consequently, the overall taxonomic composition in this site was likely more similar to SB and RB situations.

Regardless of scale, higher biodiversity often results in a larger spectrum of ecological functions on which processes and services delivered by habitats and ecosystems directly depend. Even the “simplest” ecological function depends on complex linkages between biological components^53^. Ecological functions can come from individual species (structure-forming species such as corals, molluscs, seagrasses, and others), or from the cumulative action of multiple components whose presence is influenced by different mechanisms^54^. In this sense, high-diversity assemblages are more likely to include species with hidden roles than those with lower diversity levels^55,56^. Traits such as body size, feeding behavior/diet, and mobility broadly determine what, how, and where resources are acquired, consumed, transported, and recycled. Assemblages associated with DWOR here studied were not only taxonomically rich, but taxa were also diverse in a functional perspective, reporting the highest observed values of functional richness together with C and CN assemblages. If the latter are known to host organisms with diverse ecological functions^51,52^, our results provide the first evidence that also DWOR habitats may represent hotspots of ecological functions in the Mediterranean Sea. The complex three-dimensional structures built by *N. cochlear* provide secondary hard substrates and ravines that can be exploited by sessile and vagile fauna belonging to different functional entities. Porifera, Cnidaria, and Bryozoa, indeed, colonized the dead portions of reef structure, whilst the vagile organisms were frequent in the surroundings. Similarly, the hierarchical clustering based on the functional dissimilarity grouped DWOR, C, and CN in the same cluster, suggesting that these habitats may host assemblages with high functional diversity. According to SIMPER analysis, functional entities contributing the most to the difference between the cluster DWOR-C-CN and RB-SB were those related to poriferans, solitary and colonial cnidarians and bryozoans, more abundant in DWOR, C and CN assemblages, and scavengers/predators more observed in RB and SB.

Considering solely richness and abundance, however, does not provide information on how taxa are distributed across groups, on species identity and the identification of which taxonomic groups and functions are redundant within the community^57,58^. When an assemblage presents species with similar ecological roles, this redundancy can serve as a reservoir of biological options that help ensure that an ecosystem can respond to some level of perturbation without catastrophic loss of functions^58^.

Whilst the equal distribution of individuals among taxonomic functional groups is usually interpreted as an ecologically stable situation, the dominance of some taxa and functional entities might be the result of previous or ongoing disturbances influencing the composition of the associations^59^. RB and SB reported high functional evenness with richness values significantly lower with respect to other assemblages. Here, the few functions documented were provided by different taxa, each counting many specimens. Trawling scars and lost fishing gears were indeed observed in surveys on SB and RB assemblages, suggesting that human activities were and/or are in act in the investigated sites and might have influenced the composition of the assemblages. Fauna associated with SB and RB was mainly represented by echinoderms, encrusting sponges and bryozoans and a few erected soft cnidarians colonizing the hard substrates. Previous studies have already evidenced high abundances of echinoderms in benthic habitats impacted by trawling activities, likely due to the higher potential survival capacity of high-motility taxa as well as to the increased availability of food resources for scavengers in the form of animals damaged by the fishing gears^60^. Hence, the loss of taxa may not only drive a loss of functional groups but also an increase in the evenness of those remaining.

On the contrary, a significantly lower functional evenness was observed in C, CN, and DWOR. This suggests that some functional entities were composed of a restricted number of taxa and, thus, limited in terms of biological options capable of providing the same function if taxa are lost due to perturbations.

Besides the severe alterations related to overharvesting and superseding by the alien Pacific oyster *M. gigas*, oyster reefs in the Mediterranean and Black Sea and Atlantic Ocean are now facing climate changes. Ocean acidification is of major concern for biogenic reefs, reducing the ability of organisms to calcify^9^. The Mediterranean Sea is prone to absorbing and storing anthropogenic carbon due to the particular CO_2_ chemistry, the active overturning circulation, the high evaporation rates, and the inputs from rivers and the Black Sea^61^. Hence, the Mediterranean Sea is one of the most affected regions by acidification but its high supersaturation in calcite and aragonite renders depletion in carbonate ions not a problem in the near future^62^. In addition, although effects differ among species^33^, the low-Mg calcite structure was proven to confer oysters more resistance to ocean acidification than calcifiers using other calcium carbonate forms^63^. Acidification impact, however, appears exacerbated by ocean warming, which also showed detrimentally impact on shallow-water oysters^64^, yet with species-specific responses and related to heating event duration^33^. Colonizing deeper portions of the seafloor, oyster reefs built by *N. cochlear* might be protected from, or less impacted by, thermal perturbation characterizing shallow waters, thus having a potential edge in a warming ocean.

Although scarce, literature records showed that DWOR conditions are almost pristine, with impacts mainly related to fishery and its abandoned or lost gears^36^. Conservation actions become therefore crucial to preserve the biodiversity and ecological functions associated with these overlooked reefs in the offshore. As biogenic habitats, Mediterranean DWOR are protected under the Habitats Directive^65^ but the scarcity of information has arguably led conservation measures to favor other, more studied biogenic situations. Our findings provide evidence that deep-water oyster reefs are hotspots of bio- and functional diversity in the Mediterranean Sea, representing ecologically relevant habitats that also present conditions for potential long-term survival in a changing ocean.

## Materials and methods

### Video survey collection and analyses

A total of 25 ROV surveys were collected during several oceanographic cruises performed in the framework of the Marine Strategy Framework Directive (MSFD 2008/56/CE) monitoring activities along the Italian coasts (Fig. 1; Table 1). Videos were acquired using a Pollux III (Global Electric Italiana) equipped with a low-resolution CCD video camera and a high-resolution (2304 × 1296 pixels) video camera. The ROV was provided with an underwater acoustic tracking system (USBL, Linkquest, TrackLink 1500 MA) that recorded position every second. The ROV mounted also a high-definition video camera (SONY HDR-HD7, Tokyo, Japan). Three parallel laser beams (with 20 cm separation) provided a scale during recordings.

Dive trackpoints were smoothed utilizing Adelie GIS (©Ifremer) extension for ArcGIS (© ESRI) software. The Adelie Video tool “points to line” was used to produce a line-format track of ROV dives. Frames were extracted from video recordings every 10 s using Adelie Video (© Ifremer) and analyzed for taxonomical identification. When necessary, the images were coupled with high-definition video recording to improve taxonomic identification efficiency. Macrofauna (2 mm–2 cm) and megafauna (> 2 cm) were identified to the lowest possible taxonomic rank. Organisms unidentifiable at the genus or species level were categorized as morpho-species or morphological categories^66,67^. The abundances of taxa along the ROV tracks were calculated and mapped by counting the number of taxa in each frame. To characterize the taxonomic diversity of the benthic assemblages, differences among sites were investigated in terms of taxa richness, calculated as number of benthic taxa per ROV survey and considering any taxonomic levels (species, genus, or higher taxonomic rank). Since the length of the video footages and, thus, the number of extracted frames were different, species accumulation curves representing the expected number of taxa as a function of sampling effort (number of frames) were generated using function “specaccum” of package “vegan”^48^ (version 2.5, method “random”, 1000 permutations). The value of expected taxonomic richness with 100 frames was used to compare the diversity associated with explored assemblages. Pielou’s evenness index (J), representing a measure of the relative abundances of species within a community, was calculated in R software using package “vegan” (version 2.5).

The assemblages were classified based on principal/dominant taxonomic component, identifying 5 groups:

- deep-water oyster-reef assemblages (DWOR, Fig. 2A, B): reefs built by *Neopycnodonte cochlear* populated by sponges (e.g., *Hexadella* spp. and *Axinella* spp.), bryozoans (*Schizomavella mamillata*, *Smittina cervicornis*), ascidians (*Halocynthia papillosa*) and polychaetes (*Filograna*/*Salmacina* complex);
- coralligenous assemblages (C, Fig. 2C, D): outcrops built by Corallinales algae populated by bryozoans (*Smittina cervicornis*, *Pentapora fascialis*), solitary corals (Caryophyllidae) and sponges (*Hexadella* spp. and *Axinella* spp.);
- cnidarians assemblages (CN, Fig. 2E, F): hard bottoms dominated by octocorals (e.g., *Acanthogorgia hirsuta*, *Callogorgia verticillata*) forming also dense forests, or by the scleratinian coral *Dendrophyllia cornigera*;
- rhodolith-bed assemblages (RB, Fig. 2G, H): mobile bottoms covered by living and dead non-geniculate coralline red algae characterized by the presence of echinoderms (*Stylocidaris affinis*), bryozoans (*Myriapora truncata*) and sporadic octocorals (*Eunicella singularis*);
- soft-bottom assemblages (SB, Fig. 2I, L): soft substrates mainly populated by echinoderms (e.g., *Spatangus purpureus*, *Ophiura fragilis*), soft corals (e.g., *Alcyonium palmatum*, *Pennatula rubra*) and ceriantids (*Cerianthus membranaceus*).

### Functional trait analyses

To explore the functional diversity of mesophotic assemblages, a biological trait analysis was performed on the benthic fauna associated with each site following the methods described by Mouillot et al.,^57^. The chosen functional traits focused on key characteristics, such as foraging methods, modes of locomotion, and habitat construction (Table 3). Trait assignment was categorical and coded as follows. Maximum body size (total length): meiofauna (< 2 mm), macrofauna (2 mm–2 cm) and megafauna (> 2 cm); domain of adult stage: benthic or pelagic; adult motility: sessile, facultatively motile, vagile, swimmer; feeding strategy (most frequent diet in adults): deposit feeder, grazer, filter feeder, suspension feeder, scavenger/predator, and photosynthetic metabolism; sociability (aggregation degree): solitary, gregarious, and colonial; ability to build habitat: habitat-builder, non habitat-builder.

**Table 3:** Biological traits with relative categories used for functional analysis.

Trait	Category
Adult body dimension	Meiofauna (< 2 mm)
	Macrofauna (2 mm–2 cm)
	Megafauna (> 2 cm)
	Macroalgae
Domain of adult stage	Benthic
	Pelagic
Adult motility	Sessile
	Facultatively motile
	Vagile
	Swimmer
Feeding strategy (most frequent in adult stage)	Deposit feeder
	Grazer
	Filter feeder
	Suspension feeder
	Scavenger/predator
	Photosynthetic metabolism
Sociability (aggregation degree)	Solitary
	Gregarious
	Colonial
Ability to build habitat	Habitat-builder
	Non habitat-builder

Information on biological traits related to life-cycle characteristics (reproduction, larval development, and half-life) which drive connectivity between spatially distinct populations or assemblages was not available for every taxon identified. For such a reason, these traits were not considered for the functional analysis.

Traits’ assignment was based on published accounts of the biology of each taxon, books, and websites of various scientific institutions (e.g., World Register of Marine Species (https://www.marinespecies.org), Encyclopaedia of Life (http://eol.org) databases).

Functional diversity was described using functional richness, measured as the number of unique trait value combinations in the community (Functional Entity) computed using the mFD package (v1.0.4.) in R software. The expected functional richness with 100 frames derived from accumulation curves, generated using the same methodology as for taxonomic richness, was used to compare the diversity associated with explored assemblages. Functional Evenness (FEve) for each assemblage explored were calculated by using the “mFD” package in R software.

### Statistical analyses

To exclude a potential effect of the depth on assemblages composition, the depth range associated to each taxon observed in the ROV videos was analyzed. The results showed a potential breakpoint in the composition of assemblages at 100 m depth (Fig. S1). The analyses were then performed on both the entire set of dives and dividing dives in two groups: 55–100 m and 100–200 m. The taxonomic and functional richness were tested for difference amongst different explored assemblages using one-way analysis of variance ANOVA using fixed effects, orthogonal contrasts and 5 levels when considering the entire dataset, 4 levels for 55–100 m depth range and 3 levels for 100–200 m depth range. In case of significant ANOVA, pairwise comparisons were tested using *post-hoc* Tukey’s honest significance test. The ANOVA assumptions for normal distribution and homogeneity of variance were checked using the Shapiro-Wilk test (package “stats”, version 4.2.0) and Levene’s test (package “car”, version 3.0), respectively. When the assumptions were not fulfilled, Kruskal–Wallis test and non-parametric pairwise comparisons Dunn’s test were used.

To test whether the investigated mesophotic assemblages were significantly different in the taxonomic and functional composition the ANOSIM (analysis of similarities) was performed in R software (*n* = 5 assemblages, package: vegan, version 2.5-7) considering the entire dataset.

Further insight into taxonomic and functional dissimilarities among assemblages was provided by the cluster analysis based on clustering algorithm Ward’s minimum variance method (package “stats”, version 2.15.3) on Bray-Curtis dissimilarity measures over square root transformed density data. Data were square root transformed to decrease the contribution of dominant species. The number of groups was determined using the silhouette function included in the “cluster” package representing a measure of the similarity of objects within a cluster (cohesion) rather than among clusters (separation).

The SIMPER analysis was carried out using the “vegan” package to identify functional entities contributing the most to the observed segregation from hierarchical clustering.

## Electronic supplementary material

Below is the link to the electronic supplementary material.

Supplementary Material 1

## Data Availability

All data generated during this study are included in this published article (and its supplementary information files). Data analyzed are available from the corresponding author on reasonable request.
