# Supplementary material for: Diversity and future perspectives of Mediterranean deep-water oyster reefs
Source: Sci Rep. 2024 Dec 28;14:30651. doi: 10.1038/s41598-024-77641-x (PMC11681240; doi:10.1038/s41598-024-77641-x)
Supplement: Supplementary file 1 — Supplementary Material 1 [file 41598_2024_77641_MOESM1_ESM.docx]

**Supplementary materials**

**Diversity and future perspectives of Mediterranean deep-water oyster reefs**

Giorgio Castellan^1,2*^, Lorenzo Angeletti^2,3^, Marco Taviani^1,4^

^1^ Institute of Marine Sciences, National Research Council (CNR-ISMAR), Bologna, Italy

^2^ NBFC - National Biodiversity Future Centre, Italy

^3^ Institute for Marine Biological Resources and Biotechnology, National Research Council (IRBIM-CNR), Ancona, Italy

^4^ Stazione Zoologica Anton Dohrn, Naples, Italy

Corresponding author: Giorgio Castellan; giorgio.castellan@cnr.it

**Fig S1.** Depth ranges of taxa identified in ROV dives.


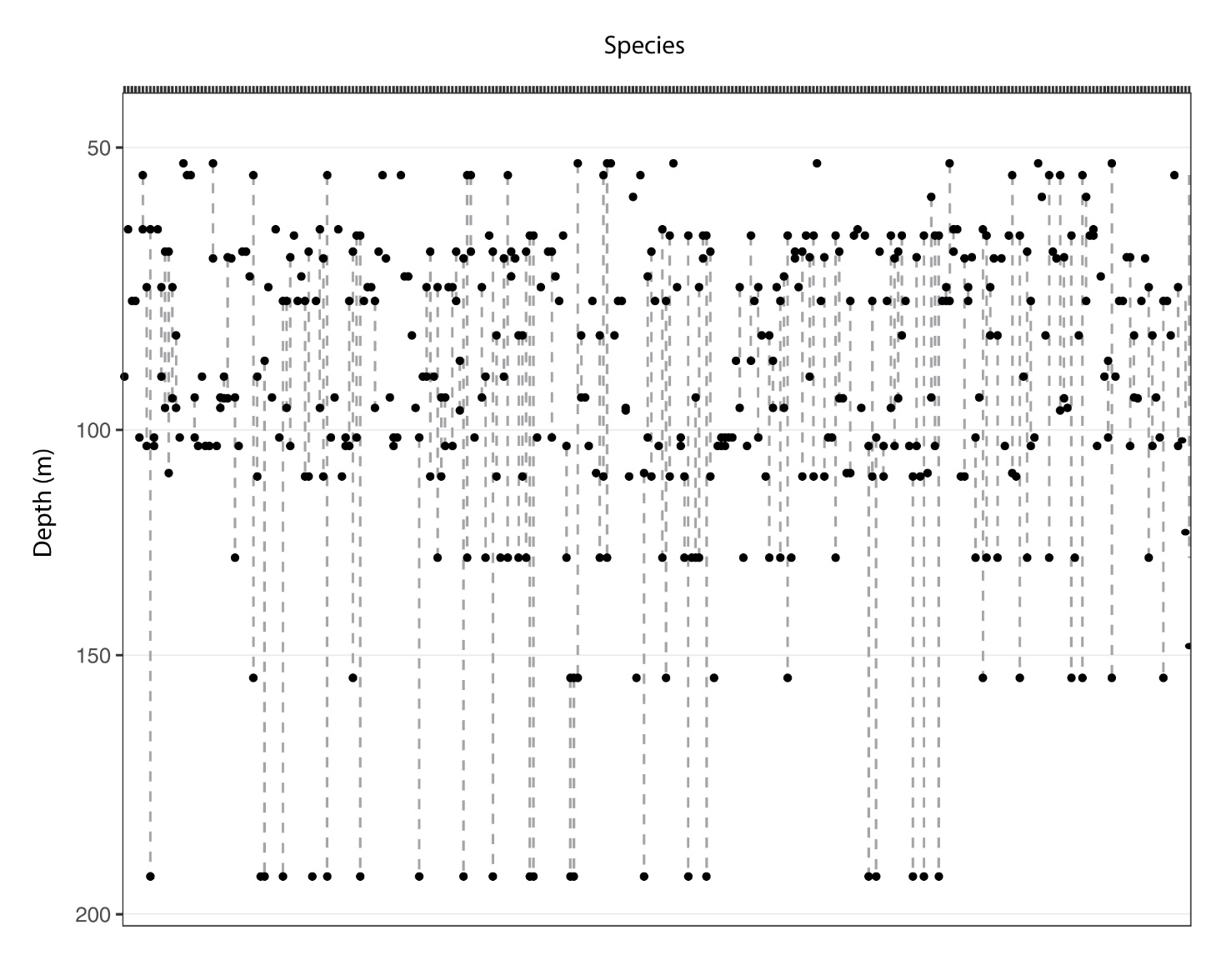


**Table S1.** Comprehensive list of the species identified during video analysis, with their abundance values and occurrence in the assemblage categories.

| Phylum | Class | Order | Family | Genus | Species | Author | ind. | CN | C | MB | DWOR | RB |
| --- | --- | --- | --- | --- | --- | --- | --- | --- | --- | --- | --- | --- |
| Rhodophyta | Florideophyceae | Corallinales |  |  | spp. | P.C. Silva & H.W. Johansen, 1986 |  | X |  |  |  | X |
| Ochrophyta | Phaeophyceae | Dictyotales | Dictyotaceae | *Dictyota* | *Dictyota dichotoma* | (Hudson) J.V. Lamouroux, 1809 | 2 |  |  |  | X |  |
| Ochrophyta | Phaeophyceae | Ectocarpales | Acinetosporaceae | *Acinetospora* | *Acinetospora crinita* | (Carmichael) Sauvageau, 1899 | 31 |  |  |  |  | X |
| Chlorophyta | Pyramimonadophyceae | Palmophyllales | Palmophyllaceae | *Palmophyllum* | *Palmophyllum crassum* | (Naccari) Rabenhorst, 1868 | 64 |  | X |  |  |  |
| Chlorophyta | Ulvophyceae | Bryopsidales | Codiaceae | *Codium* | spp. |  | 1 |  |  |  |  | X |
| Chlorophyta | Ulvophyceae | Bryopsidales | Codiaceae | *Codium* | *Codium bursa* | (Olivi) C. Agardh, 1817 | 2 |  |  |  |  | X |
| Foraminifera | Globothalamea | Rotaliida | Homotrematidae | *Miniacina* | *Miniacina miniacea* | Pallas, 1766 | 51 |  | X |  |  |  |
| Porifera | Calcarea | Clathrinida | Clathrinidae | *Clathrina* | spp. |  | 11 |  |  |  | X |  |
| Porifera | Calcarea | Clathrinida | Clathrinidae | *Clathrina* | *Clathrina blanca* | Miklucho-Maclay, 1868 | 75 |  |  |  | X |  |
| Porifera | Demospongiae | Agelasida | Agelasidae | *Agelas* | *Agelas oroides* | Schmidt, 1864 | 28 |  | X |  | X |  |
| Porifera | Demospongiae | Axinellida | Axinellidae | *Axinella* | *Axinella polypoides* | Schmidt, 1862 | 45 |  |  |  | X |  |
| Porifera | Demospongiae | Axinellida | Axinellidae | *Axinella* | sp. 1 |  | 1 |  | X |  |  |  |
| Porifera | Demospongiae | Axinellida | Axinellidae | *Axinella* | sp. 2 |  | 33 | X |  |  | X |  |
| Porifera | Demospongiae | Axinellida | Axinellidae | *Axinella* | sp. 3 |  | 2 | X |  |  |  |  |
| Porifera | Demospongiae | Axinellida | Raspailiidae | *Raspailia* | *Raspailia hispida* | Montagu, 1814 | 59 |  | X |  | X |  |
| Porifera | Demospongiae | Axinellida | Raspailiidae | *Raspailia* | *Raspailia viminalis* | Schmidt, 1862 | 6 |  |  |  | X |  |
| Porifera | Demospongiae | Bubarida | Dictyonellidae | *Dictyonella* | *Dictyonella incisa* | Schmidt, 1880 | 3 |  | X |  | X |  |
| Porifera | Demospongiae | Chondrosiida | Chondrosiidae | *Chondrosia* | *Chondrosia reniformis* | Nardo, 1847 | 67 | X | X |  | X |  |
| Porifera | Demospongiae | Clionaida | Clionaidae | *Cliona* | sp. 1 |  | 3 | X |  |  |  |  |
| Porifera | Demospongiae | Clionaida | Spirastrellidae | *Spirastrella* | *Spirastrella cunctatrix* | Schmidt, 1868 | 40 | X |  |  | X |  |
| Porifera | Demospongiae | Dictyoceratida | Dysideidae | *Dysidea* | sp. 1 |  | 34 | X | X |  |  |  |
| Porifera | Demospongiae | Dictyoceratida | Irciniidae | *Ircinia* | spp. |  | 1 | X |  |  |  |  |
| Porifera | Demospongiae | Dictyoceratida | Irciniidae | *Ircinia* | *Ircinia variabilis* | Schmidt, 1862 | 1 |  |  |  | X |  |
| Porifera | Demospongiae | Dictyoceratida | Spongiidae | *Spongia* | spp. |  | 3 | X |  |  |  | X |
| Porifera | Demospongiae | Dictyoceratida | Spongiidae | *Spongia* | *Spongia agaricina* | Pallas, 1766 | 1 |  |  |  | X |  |
| Porifera | Demospongiae | Dictyoceratida | Spongiidae | *Spongia* | *Spongia lamella* | Schulze, 1879 | 2 |  |  |  | X |  |
| Porifera | Demospongiae | Dictyoceratida | Spongiidae | *Spongia* | *Spongia officinalis* | Linnaeus, 1759 | 8 |  | X |  |  |  |
| Porifera | Demospongiae | Dictyoceratida | Thorectidae | *Scalarispongia* | spp. |  | 1 | X |  |  |  |  |
| Porifera | Demospongiae | Haplosclerida | Petrosiidae | *Petrosia* | *Petrosia ficiformis* | Poiret, 1789 | 107 | X | X |  | X |  |
| Porifera | Demospongiae | Haplosclerida | Chalinidae | *Haliclona* | *Haliclona fulva* | Topsent, 1893 | 1 |  |  |  | X |  |
| Porifera | Demospongiae | Haplosclerida | Chalinidae | *Haliclona* | sp. 1 |  | 230 | X | X |  | X |  |
| Porifera | Demospongiae | Haplosclerida | Chalinidae | *Haliclona* | sp. 2 |  | 209 | X | X |  | X |  |
| Porifera | Demospongiae | Haplosclerida | Chalinidae | *Haliclona* | sp. 3 |  | 26 |  | X |  | X |  |
| Porifera | Demospongiae | Haplosclerida | Chalinidae | *Haliclona* | sp. 4 |  | 104 | X |  |  |  |  |
| Porifera | Demospongiae | Haplosclerida | Chalinidae | *Haliclona* | sp. 5 |  | 4 | X |  |  |  |  |
| Porifera | Demospongiae | Haplosclerida | Chalinidae | *Haliclona* | sp. 6 |  | 66 | X |  |  |  |  |
| Porifera | Demospongiae | Haplosclerida | Chalinidae | *Haliclona* | sp. 7 |  | 9 | X |  |  |  |  |
| Porifera | Demospongiae | Haplosclerida | Chalinidae | *Haliclona* | sp. 8 |  | 16 | X |  |  |  |  |
| Porifera | Demospongiae | Haplosclerida | Chalinidae | *Haliclona* | sp. 9 |  | 12 |  |  |  | X |  |
| Porifera | Demospongiae | Haplosclerida | Chalinidae | *Haliclona* | sp. 10 |  | 46 |  |  |  | X |  |
| Porifera | Demospongiae | Haplosclerida | Chalinidae | *Haliclona* | sp. 11 |  | 111 | X | X |  | X |  |
| Porifera | Demospongiae | Haplosclerida | Chalinidae | *Haliclona* | sp. 12 |  | 6 |  | X |  |  |  |
| Porifera | Demospongiae | Poecilosclerida | Crambeidae | *Crambe* | spp. |  | 2 |  |  |  |  | X |
| Porifera | Demospongiae | Poecilosclerida | Crambeidae | *Crambe* | *Crambe crambe* | Schmidt, 1862 | 1 |  | X |  |  |  |
| Porifera | Demospongiae | Poecilosclerida | Esperiopsidae | *Ulosa* | *Ulosa digitata* | Schmidt, 1866 | 25 | X | X |  | X |  |
| Porifera | Demospongiae | Poecilosclerida | Hymedesmiidae | *Hemimycale* | *Hemimycale columella* | Bowerbank, 1874 | 1 |  |  |  |  | X |
| Porifera | Demospongiae | Poecilosclerida | Hymedesmiidae | *Phorbas* | *Phorbas tenacior* | Topsent, 1925 | 279 | X | X |  | X |  |
| Porifera | Demospongiae | Poecilosclerida | Microcionidae | *Clathria* | *Clathria compressa* | Schmidt, 1862 | 8 |  |  |  | X |  |
| Porifera | Demospongiae | Poecilosclerida | Mycalidae | *Mycale* | sp. 1 |  | 10 |  |  |  | X |  |
| Porifera | Demospongiae | Poecilosclerida | Tedaniidae | *Tedania* | sp. 1 |  | 18 | X |  |  |  |  |
| Porifera | Demospongiae | Polymastiida | Polymastiidae | *Polymastia* | spp. |  | 1 |  |  |  | X |  |
| Porifera | Demospongiae | Suberitida | Halichondriidae | *Halichondria* | sp. 1 |  | 342 | X |  |  |  |  |
| Porifera | Demospongiae | Suberitida | Halichondriidae | *Halichondria* | sp. 2 |  | 85 |  |  |  | X |  |
| Porifera | Demospongiae | Suberitida | Halichondriidae | *Topsentia* | *Topsentia vaceleti* | Kefalas & Castritsi-Catharios, 2012 | 6 | X |  |  | X |  |
| Porifera | Demospongiae | Suberitida | Suberitidae | *Suberites* | spp. |  | 20 |  |  |  | X |  |
| Porifera | Demospongiae | Suberitida | Suberitidae | *Suberites* | *Suberites domuncula* | Olivi, 1792 | 1 |  |  |  | X |  |
| Porifera | Demospongiae | Tethyida | Tethyidae | *Tethya* | sp. 1 |  | 1 |  |  |  | X |  |
| Porifera | Demospongiae | Tethyida | Tethyidae | *Tethya* | *Tethya aurantium* | Pallas, 1766 | 1 |  |  |  | X |  |
| Porifera | Demospongiae | Tethyida | Tethyidae | *Tethya* | *Tethya citrina* | Sarà & Melone, 1965 | 13 | X | X |  | X |  |
| Porifera | Demospongiae | Tetractinellida | Geodiidae | *Geodia* | *Geodia cydonium* | Linnaeus, 1767 | 1 |  |  |  | X |  |
| Porifera | Demospongiae | Tetractinellida | Geodiidae | *Geodia* | sp. 1 |  | 1 |  |  |  | X |  |
| Porifera | Demospongiae | Tetractinellida | Pachastrellidae | *Pachastrella* | *Pachastrella monilifera* | Schmidt, 1868 | 210 | X |  |  | X |  |
| Porifera | Demospongiae | Tetractinellida | Theneidae | *Thenea* | *Thenea muricata* | Bowerbank, 1858 | 1 |  |  |  | X |  |
| Porifera | Demospongiae | Tetractinellida | Vulcanellidae | *Poecillastra* | *Poecillastra compressa* | Bowerbank, 1866 | 245 | X |  |  | X |  |
| Porifera | Demospongiae | Verongiida | Aplysinidae | *Aplysina* | sp. 1 |  | 2 | X |  |  |  |  |
| Porifera | Demospongiae | Verongiida | Ianthellidae | *Hexadella* | *Hexadella racovitzai* | Topsent, 1896 | 255 | X | X |  | X | X |
| Porifera | Demospongiae | Verongiida | Ianthellidae | *Hexadella* | sp. 1 |  | 1 | X |  |  |  |  |
| Porifera | Demospongiae |  |  |  | sp. 1 |  | 16 |  | X |  | X |  |
| Porifera | Demospongiae |  |  |  | sp. 2 |  | 65 |  | X |  | X |  |
| Porifera | Demospongiae |  |  |  | sp. 3 |  | 1 |  | X |  |  |  |
| Porifera | Demospongiae |  |  |  | sp. 4 |  | 1852 | X | X |  | X |  |
| Porifera | Demospongiae |  |  |  | sp. 5 |  | 42 |  | X |  |  |  |
| Porifera | Demospongiae |  |  |  | sp. 6 |  | 1 |  |  |  | X |  |
| Porifera | Demospongiae |  |  |  | sp. 7 |  | 1 |  | X |  |  |  |
| Porifera | Demospongiae |  |  |  | sp. 8 |  | 3 | X |  |  | X |  |
| Porifera | Demospongiae |  |  |  | sp. 9 |  | 27 | X |  |  |  |  |
| Porifera | Demospongiae |  |  |  | sp. 10 |  | 26 | X |  |  |  |  |
| Porifera | Demospongiae |  |  |  | sp. 11 |  | 32 | X | X |  | X |  |
| Porifera | Demospongiae |  |  |  | sp. 12 |  | 18 |  |  |  | X |  |
| Porifera | Demospongiae |  |  |  | sp. 13 |  | 357 | X | X |  | X | X |
| Porifera | Demospongiae |  |  |  | sp. 14 |  | 14 |  |  |  | X |  |
| Porifera | Demospongiae |  |  |  | sp. 15 |  | 412 | X | X |  | X |  |
| Porifera | Demospongiae |  |  |  | sp. 16 |  | 17 |  | X |  |  |  |
| Porifera | Demospongiae |  |  |  | sp. 17 |  | 144 |  |  |  | X |  |
| Porifera | Demospongiae |  |  |  | sp. 18 |  | 3 |  | X |  |  |  |
| Porifera | Demospongiae |  |  |  | sp. 19 |  | 1 |  |  |  |  | X |
| Porifera | Demospongiae |  |  |  | sp. 20 |  | 50 |  | X |  | X |  |
| Porifera | Demospongiae |  |  |  | sp. 21 |  | 210 | X |  |  |  |  |
| Porifera | Demospongiae |  |  |  | sp. 22 |  | 11 |  |  |  | X |  |
| Porifera | Demospongiae |  |  |  | sp. 23 |  | 346 | X |  |  | X |  |
| Porifera | Demospongiae |  |  |  | sp. 24 |  | 1 |  |  |  | X |  |
| Porifera | Demospongiae |  |  |  | sp. 25 |  | 2 |  | X |  |  |  |
| Porifera | Demospongiae |  |  |  | sp. 26 |  | 37 |  |  |  | X |  |
| Porifera | Demospongiae |  |  |  | sp. 27 |  | 2 |  |  |  | X |  |
| Porifera | Hexactinellida | Lyssacinosida | Rossellidae | *Sympagella* | *Sympagella delauzei* | Boury-Esnault, Vacelet, Reiswig & Chevaldonné, 2015 | 3 | X |  |  |  |  |
| Porifera | Homoscleromorpha | Homosclerophorida | Plakinidae | *Corticium* | spp. |  | 1 |  |  |  |  | X |
| Porifera | Homoscleromorpha | Homosclerophorida | Plakinidae | *Corticium* | *Corticium candelabrum* | Schmidt, 1862 | 2 | X |  |  |  |  |
| Cnidaria | Anthozoa | Actiniaria | Aliciidae | *Alicia* | *Alicia mirabilis* | Johnson, 1861 | 1 |  |  |  |  | X |
| Cnidaria | Anthozoa | Actiniaria | Andresiidae | *Andresia* | *Andresia partenopea* | Andrès, 1883 | 3 |  | X | X |  |  |
| Cnidaria | Anthozoa | Actiniaria |  |  | spp. |  | 3 |  |  |  |  | X |
| Cnidaria | Anthozoa | Alcyonacea | Acanthogorgiidae | *Acanthogorgia* | *Acanthogorgia hirsuta* | Gray, 1857 | 1441 | X | X |  |  |  |
| Cnidaria | Anthozoa | Alcyonacea | Acanthogorgiidae | *Acanthogorgia* | *Acanthogorgia hirsuta* | Gray, 1857 | 2 | X |  |  |  |  |
| Cnidaria | Anthozoa | Alcyonacea | Alcyoniidae | *Alcyonium* | *Alcyonium coralloides* | Pallas, 1766 | 17 | X |  |  |  |  |
| Cnidaria | Anthozoa | Alcyonacea | Alcyoniidae | *Alcyonium* | *Alcyonium palmatum* | Pallas, 1766 | 50 |  | X | X | X |  |
| Cnidaria | Anthozoa | Alcyonacea | Coralliidae | *Corallium* | *Corallium rubrum* | Linnaeus, 1758 | 5 | X |  |  |  |  |
| Cnidaria | Anthozoa | Alcyonacea | Cornulariidae | *Cornularia* | *Cornularia cornucopiae* | Pallas, 1766 | 432 | X | X |  |  | X |
| Cnidaria | Anthozoa | Alcyonacea | Gorgoniidae | *Eunicella* | spp. |  | 7 | X |  |  |  |  |
| Cnidaria | Anthozoa | Alcyonacea | Gorgoniidae | *Eunicella* | *Eunicella cavolini* | Koch, 1887 | 6 |  |  |  | X |  |
| Cnidaria | Anthozoa | Alcyonacea | Gorgoniidae | *Eunicella* | *Eunicella singularis* | Esper, 1791 | 54 |  |  |  |  | X |
| Cnidaria | Anthozoa | Alcyonacea | Gorgoniidae | *Eunicella* | *Eunicella verrucosa* | Pallas, 1766 | 9 | X |  |  |  |  |
| Cnidaria | Anthozoa | Alcyonacea | Nidaliidae | *Nidalia* | *Nidalia studeri* | von Koch, 1891 | 7 | X |  |  |  |  |
| Cnidaria | Anthozoa | Alcyonacea | Paralcyoniidae | *Paralcyonium* | *Paralcyonium spinulosum* | Delle Chiaje, 1822 | 29 | X | X | X |  | X |
| Cnidaria | Anthozoa | Alcyonacea | Plexauridae | *Paramuricea* | *Paramuricea clavata* | Risso, 1826 | 8 |  |  |  | X |  |
| Cnidaria | Anthozoa | Alcyonacea | Plexauridae | *Paramuricea* | *Paramuricea macrospina* | Koch, 1882 | 2 | X |  |  |  |  |
| Cnidaria | Anthozoa | Alcyonacea | Plexauridae | *Swiftia* | *Swiftia pallida* | Madsen, 1970 | 63 |  | X |  |  |  |
| Cnidaria | Anthozoa | Alcyonacea | Primnoidae | *Callogorgia* | *Callogorgia verticillata* | Pallas, 1766 | 87 | X |  |  |  |  |
| Cnidaria | Anthozoa | Alcyonacea |  |  | sp. 1 |  | 12 | X |  |  |  |  |
| Cnidaria | Anthozoa | Antipatharia | Myriopathidae | *Antipathella* | *Antipathella subpinnata* | Ellis & Solander, 1786 | 3 | X |  |  |  |  |
| Cnidaria | Anthozoa | Penicillaria | Arachnactidae | *Arachnanthus* | *Arachnanthus oligopodus* | Cerfontaine, 1891 | 4 |  | X |  | X |  |
| Cnidaria | Anthozoa | Pennatulacea | Funiculinidae | *Funiculina* | *Funiculina quadrangularis* | Pallas, 1766 | 5 |  |  | X | X |  |
| Cnidaria | Anthozoa | Pennatulacea | Pennatulidae | *Pennatula* | *Pennatula phosphorea* | Linnaeus, 1758 | 19 | X |  |  |  | X |
| Cnidaria | Anthozoa | Pennatulacea | Pennatulidae | *Pennatula* | *Pennatula rubra* | Ellis, 1761 | 167 |  |  | X | X |  |
| Cnidaria | Anthozoa | Pennatulacea | Virgulariidae | *Virgularia* | *Virgularia mirabilis* | Müller, 1776 | 2 |  | X |  |  |  |
| Cnidaria | Anthozoa | Scleractinia | Caryophylliidae | *Caryophyllia* | sp. 1 |  | 197 | X | X |  | X |  |
| Cnidaria | Anthozoa | Scleractinia | Caryophylliidae | *Caryophyllia* | sp. 2 |  | 152 | X | X |  | X |  |
| Cnidaria | Anthozoa | Scleractinia | Caryophylliidae |  | spp. | Lamarck, 1801 | 2 |  | X |  |  |  |
| Cnidaria | Anthozoa | Scleractinia | Caryophylliidae |  | sp. 1 |  | 938 | X | X |  | X |  |
| Cnidaria | Anthozoa | Scleractinia | Caryophylliidae |  | sp. 2 |  | 85 |  |  |  | X |  |
| Cnidaria | Anthozoa | Scleractinia | Dendrophylliidae | *Balanophyllia* | *Balanophyllia europaea* | Risso, 1826 | 1 |  | X |  |  |  |
| Cnidaria | Anthozoa | Scleractinia | Dendrophylliidae | *Balanophyllia* | sp. 1 |  | 2 |  |  |  | X |  |
| Cnidaria | Anthozoa | Scleractinia | Dendrophylliidae | *Dendrophyllia* | *Dendrophyllia cornigera* | Lamarck, 1816 | 148 | X |  |  | X |  |
| Cnidaria | Anthozoa | Spirularia | Cerianthidae | *Cerianthus* | spp. | Delle Chiaje, 1841 | 1 |  | X |  |  |  |
| Cnidaria | Anthozoa | Spirularia | Cerianthidae | *Cerianthus* | *Cerianthus membranaceus* | Gmelin, 1791 | 41 |  | X | X | X | X |
| Cnidaria | Anthozoa | Spirularia | Cerianthidae |  | spp. |  | 3 |  |  |  | X |  |
| Cnidaria | Anthozoa | Spirularia | Cerianthidae |  | sp. 1 |  | 2 |  | X |  |  |  |
| Cnidaria | Anthozoa | Zoantharia | Epizoanthidae | *Epizoanthus* | sp. 1 |  | 16 | X | X |  |  |  |
| Cnidaria | Anthozoa | Zoantharia | Epizoanthidae | *Epizoanthus* | sp. 2 |  | 2 |  |  |  |  | X |
| Cnidaria | Anthozoa | Zoantharia | Parazoanthidae | *Parazoanthus* | sp. 1 |  | 22 |  | X |  | X |  |
| Cnidaria | Hydrozoa | Leptothecata | Aglaopheniidae | *Aglaophenia* | sp. 1 |  | 24 | X |  |  | X |  |
| Cnidaria | Hydrozoa | Leptothecata | Aglaopheniidae | *Lytocarpia* | *Lytocarpia myriophyllum* | Linnaeus, 1758 | 105 |  |  | X | X |  |
| Cnidaria | Hydrozoa | Leptothecata | Plumulariidae | *Nemertesia* | spp. | Lamouroux, 1812 | 30 | X | X |  | X |  |
| Mollusca | Bivalvia | Cardiida | Cardiidae | *Acanthocardia* | *Acanthocardia aculeata* | Linnaeus, 1758 | 3 |  |  |  | X |  |
| Mollusca | Bivalvia | Cardiida | Cardiidae | *Acanthocardia* | sp. 1 |  | 1 |  |  |  | X |  |
| Mollusca | Bivalvia | Ostreida | Gryphaeidae | *Neopycnodonte* | *Neopycnodonte cochlear* | Poli, 1795 | 115 |  |  | X | X | X |
| Mollusca | Bivalvia | Ostreida | Pteriidae | *Pteria* | *Pteria hirundo* | Linnaeus, 1758 | 2 |  | X | X |  |  |
| Mollusca | Bivalvia | Pectinida | Pectinidae | *Pecten* | spp. |  | 1 |  |  |  |  | X |
| Mollusca | Cephalopoda | Octopoda | Eledonidae | *Eledone* | *Eledone cirrhosa* | Lamarck, 1798 | 4 |  |  | X |  |  |
| Mollusca | Cephalopoda | Octopoda | Octopodidae | *Callistoctopus* | *Callistoctopus macropus* | Risso, 1826 | 1 |  |  |  | X |  |
| Mollusca | Cephalopoda | Octopoda | Octopodidae | *Octopus* | *Octopus vulgaris* | Cuvier, 1797 | 2 |  |  | X | X |  |
| Mollusca | Cephalopoda | Sepiida | Sepiidae | *Sepia* | spp. |  | 2 | X |  | X |  |  |
| Mollusca | Gastropoda | Littorinimorpha | Cypraeidae | *Naria* | spp. |  | 3 | X |  |  |  |  |
| Mollusca | Gastropoda | Neogastropoda | Columbellidae | *Columbella* | *Columbella rustica* | Linnaeus, 1758 | 1 |  |  |  |  | X |
| Mollusca | Gastropoda | Neogastropoda | Fasciolariidae | *Fusinus* | spp. |  | 1 |  |  |  | X |  |
| Mollusca | Gastropoda | Neogastropoda | Fasciolariidae | *Tarantinaea* | spp. |  | 1 |  |  |  | X |  |
| Mollusca | Gastropoda | Neogastropoda | Muricidae | *Hexaplex* | *Hexaplex trunculus* | Linnaeus, 1758 | 1 |  |  |  | X |  |
| Mollusca | Gastropoda | Nudibranchia | Chromodorididae | *Felimare* | *Felimare tricolor* | Cantraine, 1835 | 4 |  |  |  | X | X |
| Mollusca | Gastropoda | Nudibranchia | Discodorididae | *Discodoris* | spp. |  | 5 |  | X |  |  |  |
| Mollusca | Gastropoda | Nudibranchia | Discodorididae | *Platydoris* | *Platydoris argo* | Linnaeus, 1767 | 2 |  | X |  |  |  |
| Mollusca | Gastropoda | Nudibranchia | Myrrhinidae | *Dondice* | *Dondice banyulensis* | Portmann & Sandmeier, 1960 | 1 |  |  |  | X |  |
| Mollusca | Gastropoda | Pleurobranchida | Pleurobranchidae | *Pleurobranchus* | *Pleurobranchus testudinarius* | Cantraine, 1835 | 1 |  |  |  | X |  |
| Mollusca | Gastropoda | Trochida | Calliostomatidae | *Calliostoma* | spp. |  | 2 | X |  |  | X |  |
| Mollusca | Gastropoda | Trochida | Turbinidae | *Bolma* | *Bolma rugosa* | Linnaeus, 1767 | 1 |  | X |  |  |  |
| Mollusca | Gastropoda |  | Plakobranchidae | *Elysia* | sp. 1 |  | 1 |  | X |  |  |  |
| Mollusca | Gastropoda |  |  |  | spp. | " |  |  |  |  |  |  |
| Mollusca | Gastropoda |  |  |  | sp. 1 |  |  |  |  |  |  |  |
| Mollusca | Gastropoda |  |  |  | sp. 2 |  | 3 |  |  |  | X |  |
| Annelida | Polychaeta | Echiuroidea | Bonelliidae | *Bonellia* | *Bonellia viridis* |  | 1 |  |  |  | X |  |
| Annelida | Polychaeta | Sabellida | Sabellidae | *Sabella* | spp. | Rolando, 1822 | 58 | X | X |  | X | X |
| Annelida | Polychaeta | Sabellida | Sabellidae | *Sabella* | *Sabella pavonina* | Linnaeus, 1767 | 7 |  |  | X | X |  |
| Annelida | Polychaeta | Sabellida | Sabellidae | *Sabella* | sp. 1 | Savigny, 1822 | 111 |  | X | X | X | X |
| Annelida | Polychaeta | Sabellida | Sabellidae |  | spp. |  | 37 | X | X | X | X | X |
| Annelida | Polychaeta | Sabellida | Sabellidae |  | sp. 1 | Latreille, 1825 | 5 |  | X |  |  | X |
| Annelida | Polychaeta | Sabellida | Serpulidae | *Filograna* | *Filograna implexa* |  | 2 |  | X |  |  |  |
| Annelida | Polychaeta | Sabellida | Serpulidae | *Protula* | sp. 1 | Berkeley, 1835 | 395 | X | X | X | X |  |
| Annelida | Polychaeta | Sabellida | Serpulidae | *Serpula* | *Serpula vermicularis* |  | 110 | X | X |  | X | X |
| Annelida | Polychaeta | Sabellida | Serpulidae | *Vermiliopsis* | sp. 1 | Linnaeus, 1767 | 1 |  |  |  | X |  |
| Annelida | Polychaeta | Sabellida | Serpulidae |  | spp. |  | 4 |  | X |  | X |  |
| Annelida | Polychaeta | Terebellida | Terebellidae | *Lanice* | *Lanice conchilega* | Rafinesque, 1815 | 9 | X | X | X | X |  |
| Annelida | Polychaeta | Terebellida | Trichobranchidae | *Terebellides* | sp. 1 | Pallas, 1766 | 48 | X | X |  | X |  |
| Arthropoda | Malacostraca | Decapoda | Calappidae | *Calappa* | *Calappa granulata* |  | 7 | X |  |  | X |  |
| Arthropoda | Malacostraca | Decapoda | Inachidae | *Inachus* | *Inachus phalangium* | Linnaeus, 1758 | 1 |  |  |  | X |  |
| Arthropoda | Malacostraca | Decapoda | Inachidae | *Macropodia* | *Macropodia longirostris* | Fabricius, 1775 | 7 |  | X | X | X |  |
| Arthropoda | Malacostraca | Decapoda | Munididae | *Munida* | spp. | Fabricius, 1775 | 1 |  |  |  | X |  |
| Arthropoda | Malacostraca | Decapoda | Munididae | *Munida* | *Munida tenuimana* |  | 11 |  | X |  | X |  |
| Arthropoda | Malacostraca | Decapoda | Paguridae | *Paguristes* | *Paguristes longirostris* | Sars, 1872 | 8 |  | X |  |  |  |
| Arthropoda | Malacostraca | Decapoda | Paguridae |  | spp. | Dana, 1851 | 1 |  |  |  | X |  |
| Arthropoda | Malacostraca | Decapoda | Paguridae |  | sp. 1 | Latreille, 1802 | 4 |  | X | X | X |  |
| Arthropoda | Malacostraca | Decapoda | Paguridae |  | sp. 2 |  | 3 |  |  |  | X |  |
| Arthropoda | Malacostraca | Decapoda | Paguridae |  | sp. 3 |  | 3 |  | X |  |  | X |
| Arthropoda | Malacostraca | Decapoda | Paguridae |  | sp. 4 |  | 1 |  |  |  |  | X |
| Arthropoda | Malacostraca | Decapoda | Palinuridae | *Palinurus* | *Palinurus elephas* |  | 2 |  |  |  | X |  |
| Arthropoda | Malacostraca | Decapoda | Parthenopidae | *Spinolambrus* | spp. | Fabricius, 1787 | 7 | X | X |  | X |  |
| Arthropoda | Malacostraca | Decapoda | Polybiidae | *Liocarcinus* | spp. |  | 1 |  |  |  |  | X |
| Arthropoda | Malacostraca | Decapoda | Polybiidae | *Liocarcinus* | *Liocarcinus depurator* |  | 2 |  | X |  |  |  |
| Arthropoda | Malacostraca | Decapoda |  |  | spp. | Linnaeus, 1758 | 1 |  |  |  | X |  |
| Arthropoda | Malacostraca | Decapoda |  |  | sp. 1 | Latreille, 1802 | 2 |  |  |  | X |  |
| Arthropoda | Malacostraca | Decapoda |  |  | sp. 2 |  | 2 | X |  |  |  |  |
| Arthropoda | Malacostraca | Euphausiacea |  |  | spp. | Latreille, 1802 | 13 | X |  | X | X |  |
| Bryozoa | Gymnolaemata | Cheilostomatida | Adeonidae | *Adeonella* | *Adeonella calveti* |  | 1 |  |  |  | X |  |
| Bryozoa | Gymnolaemata | Cheilostomatida | Bitectiporidae | *Pentapora* | *Pentapora fascialis* | Canu & Bassler, 1930 | 2 |  |  |  | X |  |
| Bryozoa | Gymnolaemata | Cheilostomatida | Bitectiporidae | *Schizomavella* | *Schizomavella linearis* | Pallas, 1766 | 80 |  | X |  | X |  |
| Bryozoa | Gymnolaemata | Cheilostomatida | Bitectiporidae | *Schizomavella* | *Schizomavella mamillata* | Hassall, 1841 | 1 |  |  |  |  | X |
| Bryozoa | Gymnolaemata | Cheilostomatida | Bitectiporidae | *Schizomavella* | sp. 1 | Hincks, 1880 | 2076 |  | X |  | X | X |
| Bryozoa | Gymnolaemata | Cheilostomatida | Bugulidae | *Bugula* | spp. |  | 2 |  |  |  | X |  |
| Bryozoa | Gymnolaemata | Cheilostomatida | Celleporidae | *Turbicellepora* | *Turbicellepora avicularis* |  | 1 |  | X |  |  |  |
| Bryozoa | Gymnolaemata | Cheilostomatida | Myriaporidae | *Myriapora* | spp. | Hincks, 1860 | 1 |  | X |  |  |  |
| Bryozoa | Gymnolaemata | Cheilostomatida | Myriaporidae | *Myriapora* | *Myriapora truncata* |  | 4 |  |  |  | X |  |
| Bryozoa | Gymnolaemata | Cheilostomatida | Phidoloporidae | *Reteporella* | spp. | Pallas, 1766 | 16 |  | X |  | X | X |
| Bryozoa | Gymnolaemata | Cheilostomatida | Phidoloporidae | *Reteporella* | *Reteporella grimaldii* | Busk, 1884 | 2 | X |  |  |  |  |
| Bryozoa | Gymnolaemata | Cheilostomatida | Phidoloporidae | *Reteporella* | sp. 1 | Jullien, 1903 | 42 | X | X |  | X |  |
| Bryozoa | Gymnolaemata | Cheilostomatida | Smittinidae | *Smittina* | *Smittina cervicornis* |  | 2 |  | X |  |  |  |
| Bryozoa | Stenolaemata | Cyclostomatida | Horneridae | *Hornera* | *Hornera frondiculata* | Pallas, 1766 | 296 | X | X |  | X |  |
| Echinodermata | Asteroidea | Forcipulatida | Asteriidae | *Coscinasterias* | *Coscinasterias tenuispina* | Lamarck, 1816 | 61 |  | X |  | X |  |
| Echinodermata | Asteroidea | Forcipulatida | Asteriidae | *Marthasterias* | *Marthasterias glacialis* | Lamarck, 1816 | 1 |  |  |  | X |  |
| Echinodermata | Asteroidea | Paxillosida | Astropectinidae | *Astropecten* | spp. | Linnaeus, 1758 | 8 |  |  |  | X | X |
| Echinodermata | Asteroidea | Paxillosida | Astropectinidae | *Astropecten* | *Astropecten aranciacus* |  | 3 |  |  | X |  | X |
| Echinodermata | Asteroidea | Paxillosida | Luidiidae | *Luidia* | *Luidia ciliaris* | Linnaeus, 1758 | 1 |  |  |  |  | X |
| Echinodermata | Asteroidea | Spinulosida | Echinasteridae | *Echinaster* | *Echinaster sepositus* | Philippi, 1837 | 1 |  |  |  | X |  |
| Echinodermata | Asteroidea | Valvatida | Chaetasteridae | *Chaetaster* | *Chaetaster longipes* | Retzius, 1783 | 45 | X | X |  | X | X |
| Echinodermata | Asteroidea | Valvatida | Goniasteridae | *Peltaster* | *Peltaster placenta* | Bruzelius, 1805 | 5 | X |  |  | X |  |
| Echinodermata | Asteroidea | Valvatida | Ophidiasteridae | *Hacelia* | *Hacelia attenuata* | Müller & Troschel, 1842 | 42 | X | X |  | X |  |
| Echinodermata | Crinoidea | Comatulida | Antedonidae | *Antedon* | *Antedon mediterranea* | Gray, 1840 | 5 | X |  |  |  |  |
| Echinodermata | Crinoidea | Comatulida | Antedonidae | *Leptometra* | *Leptometra phalangium* | Lamarck, 1816 | 127 | X | X |  | X | X |
| Echinodermata | Echinoidea | Camarodonta | Echinidae | *Echinus* | *Echinus melo* | Müller, 1841 | 11 |  |  | X | X |  |
| Echinodermata | Echinoidea | Cidaroida | Cidaridae | *Cidaris* | *Stylocidaris affinis* | Lamarck, 1816 | 12 | X | X |  |  |  |
| Echinodermata | Echinoidea | Cidaroida | Cidaridae | *Cidaris* | *Cidaris cidaris* | Philippi, 1845 | 751 | X | X | X |  | X |
| Echinodermata | Echinoidea | Diadematoida | Diadematidae | *entrostephanus* | *Centrostephanus longispinus* | Linnaeus, 175 | 59 | X |  | X | X |  |
| Echinodermata | Echinoidea | Holothuriida | Holothuriidae | *Holothuria* | spp. | Philippi, 1845 | 14 |  | X |  | X | X |
| Echinodermata | Echinoidea | Holothuriida | Holothuriidae | *Holothuria* | *Holothuria forskali* |  | 1 |  |  | X |  |  |
| Echinodermata | Echinoidea | Holothuriida | Holothuriidae | *Holothuria* | *Holothuria poli* | Delle Chiaje, 1823 | 5 | X |  |  | X | X |
| Echinodermata | Echinoidea | Holothuriida | Holothuriidae | *Holothuria* | *Holothuria tubulosa* | Delle Chiaje, 1824 | 5 | X |  |  | X |  |
| Echinodermata | Echinoidea | Spatangoida | Brissidae | *Brissus* | *Brissus unicolor* | Gmelin, 1788 | 2 |  | X |  |  |  |
| Echinodermata | Echinoidea | Spatangoida | Spatangidae | *Spatangus* | *Spatangus purpureus* | Leske, 1778 | 1 |  |  |  |  | X |
| Echinodermata | Holothuroidea | Dendrochirotida | Cucumariidae | *Cucumaria* | sp. 1 | O.F. Müller, 1776 | 82 |  |  | X |  | X |
| Echinodermata | Holothuroidea | Holothuriida | Mesothuriidae | *Mesothuria* | spp. |  | 2 |  |  |  | X |  |
| Echinodermata | Holothuroidea | Synallactida | Stichopodidae | *Parastichopus* | *Parastichopus regalis* |  | 1 |  | X |  |  |  |
| Echinodermata | Ophiuroidea | Amphilepidida | Ophiopsilidae | *Ophiopsila* | spp. | Cuvier, 1817 | 1 |  |  | X |  |  |
| Echinodermata | Ophiuroidea | Amphilepidida | Ophiotrichidae | *Ophiothrix* | *Ophiothrix fragilis* |  | 2 |  |  |  |  | X |
| Echinodermata | Ophiuroidea | Euryalida | Gorgonocephalidae | *Astrospartus* | *Astrospartus mediterraneus* | Abildgaard in O.F. Müller, 1789 | 890 |  | X | X | X | X |
| Echinodermata | Ophiuroidea | Ophiacanthida | Ophiodermatidae | *Ophioderma* | spp. | Risso, 1826 | 5 | X |  |  |  |  |
| Echinodermata | Ophiuroidea | Ophiacanthida | Ophiomyxidae | *Ophiomyxa* | *Ophiomyxa pentagona* |  | 1 |  | X |  |  |  |
| Echinodermata | Ophiuroidea |  |  |  | sp. 1 | Lamarck, 1816 | 1 |  |  | X |  |  |
| Chordata | Actinopterygii | Anguilliformes | Congridae | *Ariosoma* | *Ariosoma balearicum* |  | 35 |  |  |  | X |  |
| Chordata | Actinopterygii | Anguilliformes | Muraenidae | *Muraena* | *Muraena helena* | Delaroche, 1809 | 3 |  |  |  | X |  |
| Chordata | Actinopterygii | Anguilliformes | Ophichthidae | *Ophisurus* | *Ophisurus serpens* | Linnaeus, 1758 | 23 | X | X |  | X |  |
| Chordata | Actinopterygii | Anguilliformes |  |  | spp. | Linnaeus, 1758 | 1 |  |  | X |  |  |
| Chordata | Actinopterygii | Gadiformes | Phycidae | *Phycis* | spp. |  | 6 |  |  |  | X |  |
| Chordata | Actinopterygii | Gadiformes | Phycidae | *Phycis* | *Phycis blennoides* |  | 3 | X |  |  | X |  |
| Chordata | Actinopterygii | Gadiformes | Phycidae | *Phycis* | *Phycis phycis* | Brünnich, 1768 | 3 | X |  |  |  |  |
| Chordata | Actinopterygii | Perciformes | Callanthiidae | *Callanthias* | *Callanthias ruber* | Linnaeus, 1766 | 1 |  |  |  | X |  |
| Chordata | Actinopterygii | Perciformes | Gobiidae |  | spp. | Rafinesque, 1810 | 161 | X | X |  | X | X |
| Chordata | Actinopterygii | Perciformes | Labridae | *Ctenolabrus* | *Ctenolabrus rupestris* |  | 9 |  |  | X |  |  |
| Chordata | Actinopterygii | Perciformes | Labridae | *Lappanella* | *Lappanella fasciata* | Linnaeus, 1758 | 2 |  |  |  | X |  |
| Chordata | Actinopterygii | Perciformes | Mullidae | *Mullus* | spp. | Cocco, 1833 | 2 |  |  |  | X |  |
| Chordata | Actinopterygii | Perciformes | Mullidae | *Mullus* | *Mullus barbatus* |  | 2 |  |  |  | X | X |
| Chordata | Actinopterygii | Perciformes | Pomacentridae | *Chromis* | spp. | Linnaeus, 1758 | 4 |  |  |  | X |  |
| Chordata | Actinopterygii | Perciformes | Scorpaenidae | *Scorpaena* | *Scorpaena scrofa* |  | 43 |  |  |  |  | X |
| Chordata | Actinopterygii | Perciformes | Serranidae | *Serranus* | spp. | Linnaeus, 1758 | 21 | X | X | X | X |  |
| Chordata | Actinopterygii | Perciformes | Serranidae | *Serranus* | *Serranus cabrilla* |  | 3 |  |  |  | X |  |
| Chordata | Actinopterygii | Perciformes | Serranidae | *Serranus* | *Serranus hepatus* | Linnaeus, 1758 | 31 | X | X | X | X | X |
| Chordata | Actinopterygii | Perciformes | Serranidae |  | sp. 1 | Linnaeus, 1758 | 4 |  |  | X |  |  |
| Chordata | Actinopterygii | Perciformes | Sparidae | *Pagellus* | spp. |  | 2 |  |  |  | X |  |
| Chordata | Actinopterygii | Perciformes | Sparidae | *Pagellus* | *Pagellus erythrinus* |  | 5 |  | X |  | X |  |
| Chordata | Actinopterygii | Perciformes | Sparidae | *Spondyliosoma* | *Spondyliosoma cantharus* | Linnaeus, 1758 | 1 |  |  |  | X |  |
| Chordata | Actinopterygii | Perciformes | Sparidae |  | spp. | Linnaeus, 1758 | 1 |  |  |  | X |  |
| Chordata | Actinopterygii | Perciformes | Synodontidae | *Synodus* | *Synodus saurus* |  | 1 |  |  |  | X |  |
| Chordata | Actinopterygii | Perciformes | Trachinidae | *Trachinus* | spp. | Linnaeus, 1758 | 1 |  |  |  | X |  |
| Chordata | Actinopterygii | Perciformes | Trachinidae | *Trachinus* | *Trachinus araneus* |  | 1 |  |  |  |  | X |
| Chordata | Actinopterygii | Perciformes | Triglidae | *Eutrigla* | sp. 1 | Cuvier, 1829 | 2 |  |  | X |  |  |
| Chordata | Actinopterygii | Pleuronectiformes | Soleidae | *Solea* | *Solea solea* |  | 1 |  |  | X |  |  |
| Chordata | Actinopterygii | Scorpaeniformes | Sebastidae | *Helicolenus* | *Helicolenus dactylopterus* | Linnaeus, 1758 | 1 |  |  |  | X |  |
| Chordata | Actinopterygii | Scorpaeniformes | Triglidae | *Chelidonichthys* | *Chelidonichthys lastoviza* | Delaroche, 1809 | 17 | X |  | X |  |  |
| Chordata | Actinopterygii | Scorpaeniformes | Triglidae | *Chelidonichthys* | *Chelidonichthys lucerna* | Bonnaterre, 1788 | 8 |  |  |  |  | X |
| Chordata | Actinopterygii | Scorpaeniformes | Triglidae | *Lepidotrigla* | *Lepidotrigla cavillone* | Linnaeus, 1758 | 1 | X |  |  |  |  |
| Chordata | Actinopterygii | Zeiformes | Zeidae | *Zeus* | *Zeus faber* | Lacepède, 1801 | 3 |  | X | X |  |  |
| Chordata | Actinopterygii |  |  |  | sp. 1 | Linnaeus, 1758 | 4 | X |  |  | X | X |
| Chordata | Ascidiacea | Aplousobranchia | Clavelinidae | *Clavelina* | sp. 1 |  | 15 |  |  |  |  | X |
| Chordata | Ascidiacea | Aplousobranchia | Diazonidae | *Rhopalaea* | spp. |  | 4 | X |  |  |  |  |
| Chordata | Ascidiacea | Aplousobranchia | Diazonidae | *Rhopalaea* | *Rhopalaea neapolitana* |  | 7 |  | X |  |  |  |
| Chordata | Ascidiacea | Aplousobranchia | Didemnidae | *Diplosoma* | *Diplosoma spongiforme* | Philippi, 1843 | 4 |  | X |  |  |  |
| Chordata | Ascidiacea | Aplousobranchia | Didemnidae | *Polysyncraton* | *Polysyncraton lacazei* | Giard, 1872 | 9 |  | X |  | X |  |
| Chordata | Ascidiacea | Aplousobranchia | Didemnidae |  | sp. 1 | Giard, 1872 | 4 |  | X |  | X |  |
| Chordata | Ascidiacea | Aplousobranchia | Didemnidae |  | sp. 2 |  | 41 |  | X |  | X |  |
| Chordata | Ascidiacea | Aplousobranchia | Didemnidae |  | sp. 3 |  | 6 |  |  |  | X |  |
| Chordata | Ascidiacea | Aplousobranchia | Polyclinidae | *Aplidium* | sp. 1 |  | 18 | X |  |  | X |  |
| Chordata | Ascidiacea | Phlebobranchia | Cionidae | *Ciona* | spp. |  | 4 | X |  |  |  |  |
| Chordata | Ascidiacea | Stolidobranchia | Pyuridae | *Halocynthia* | *Halocynthia papillosa* |  | 3 |  | X |  |  |  |
| Chordata | Ascidiacea |  |  |  | spp. | Linnaeus, 1767 | 66 | X | X |  | X | X |
| Chordata | Ascidiacea |  |  |  | sp. 1 |  | 1 |  |  | X | X |  |
| Chordata | Ascidiacea |  |  |  | sp. 2 |  | 8 |  | X |  | X |  |
| Chordata | Ascidiacea |  |  |  | sp. 3 |  | 5 | X |  |  |  |  |
| Chordata | Ascidiacea |  |  |  | sp. 4 |  | 6 |  |  |  | X |  |

**Tab. S2.** Classification of identified taxa using functional traints. NHB: non habitat builder; HB: habitat builder.

| Phylum | **Class** | **Order** | **Family** | **Genus** | **Species** | **Adult dimension** | **Domain** | **Adult motility** | **Feeding strategy** | **Sociability** | **Ability to build habitat** |
| --- | --- | --- | --- | --- | --- | --- | --- | --- | --- | --- | --- |
| Chlorophyta | Pyramimonadophyceae | Palmophyllales | Palmophyllaceae | *Palmophyllum* | *Palmophyllum crassum* | Macroalgae | Benthic | Sessile | Photosynthetic | Solitary | NHB |
| Chlorophyta | Ulvophyceae | Bryopsidales | Codiaceae | *Codium* | spp. | Macroalgae | Benthic | Sessile | Photosynthetic | Solitary | NHB |
| Chlorophyta | Ulvophyceae | Bryopsidales | Codiaceae | *Codium* | *Codium bursa* | Macroalgae | Benthic | Sessile | Photosynthetic | Solitary | NHB |
| Ochrophyta | Phaeophyceae | Dictyotales | Dictyotaceae | *Dictyota* | *Dictyota dichotoma* | Macroalgae | Benthic | Sessile | Photosynthetic | Solitary | NHB |
| Ochrophyta | Phaeophyceae | Ectocarpales | Acinetosporaceae | *Acinetospora* | *Acinetospora crinita* | Macroalgae | Benthic | Sessile | Photosynthetic | Solitary | NHB |
| Rhodophyta | Florideophyceae | Corallinales |  |  | spp. | Macroalgae | Benthic | Sessile | Photosynthetic | Solitary | NHB |
| Foraminifera | Globothalamea | Rotaliida | Homotrematidae | *Miniacina* | *Miniacina miniacea* | Macrofauna | Benthic | Vagile | Scavenger/Predator | Solitary | NHB |
| Porifera | Calcarea | Clathrinida | Clathrinidae | *Clathrina* | *Clathrina blanca* | Megafauna | Benthic | Sessile | Filter feeder | Solitary | NHB |
| Porifera | Calcarea | Clathrinida | Clathrinidae | *Clathrina* | spp. | Megafauna | Benthic | Sessile | Filter feeder | Solitary | NHB |
| Porifera | Demospongiae | Agelasida | Agelasidae | *Agelas* | *Agelas oroides* | Megafauna | Benthic | Sessile | Filter feeder | Solitary | NHB |
| Porifera | Demospongiae | Axinellida | Axinellidae | *Axinella* | *Axinella polypoides* | Megafauna | Benthic | Sessile | Filter feeder | Solitary | HB |
| Porifera | Demospongiae | Axinellida | Axinellidae | *Axinella* | sp. 1 | Megafauna | Benthic | Sessile | Filter feeder | Solitary | HB |
| Porifera | Demospongiae | Axinellida | Axinellidae | *Axinella* | sp. 2 | Megafauna | Benthic | Sessile | Filter feeder | Solitary | HB |
| Porifera | Demospongiae | Axinellida | Axinellidae | *Axinella* | sp. 3 | Megafauna | Benthic | Sessile | Filter feeder | Solitary | HB |
| Porifera | Demospongiae | Axinellida | Raspailiidae | *Raspailia* | *Raspailia hispida* | Megafauna | Benthic | Sessile | Filter feeder | Solitary | NHB |
| Porifera | Demospongiae | Axinellida | Raspailiidae | *Raspailia* | *Raspailia viminalis* | Megafauna | Benthic | Sessile | Filter feeder | Solitary | NHB |
| Porifera | Demospongiae | Bubarida | Dictyonellidae | *Dictyonella* | *Dictyonella incisa* | Megafauna | Benthic | Sessile | Filter feeder | Solitary | NHB |
| Porifera | Demospongiae | Chondrosiida | Chondrosiidae | *Chondrosia* | *Chondrosia reniformis* | Megafauna | Benthic | Sessile | Filter feeder | Solitary | NHB |
| Porifera | Demospongiae | Clionaida | Clionaidae | *Cliona* | sp. 1 | Megafauna | Benthic | Sessile | Filter feeder | Solitary | NHB |
| Porifera | Demospongiae | Clionaida | Spirastrellidae | *Spirastrella* | *Spirastrella cunctatrix* | Megafauna | Benthic | Sessile | Filter feeder | Solitary | NHB |
| Porifera | Demospongiae | Dictyoceratida | Dysideidae | *Dysidea* | sp. 1 | Megafauna | Benthic | Sessile | Filter feeder | Solitary | NHB |
| Porifera | Demospongiae | Dictyoceratida | Irciniidae | *Ircinia* | *Ircinia variabilis* | Megafauna | Benthic | Sessile | Filter feeder | Solitary | NHB |
| Porifera | Demospongiae | Dictyoceratida | Irciniidae | *Ircinia* | spp. | Megafauna | Benthic | Sessile | Filter feeder | Solitary | NHB |
| Porifera | Demospongiae | Dictyoceratida | Spongiidae | *Spongia* | spp. | Megafauna | Benthic | Sessile | Filter feeder | Solitary | NHB |
| Porifera | Demospongiae | Dictyoceratida | Spongiidae | *Spongia* | *Spongia agaricina* | Megafauna | Benthic | Sessile | Filter feeder | Solitary | NHB |
| Porifera | Demospongiae | Dictyoceratida | Spongiidae | *Spongia* | *Spongia lamella* | Megafauna | Benthic | Sessile | Filter feeder | Solitary | NHB |
| Porifera | Demospongiae | Dictyoceratida | Spongiidae | *Spongia* | *Spongia officinalis* | Megafauna | Benthic | Sessile | Filter feeder | Solitary | NHB |
| Porifera | Demospongiae | Dictyoceratida | Thorectidae | *Scalarispongia* | spp. | Megafauna | Benthic | Sessile | Filter feeder | Solitary | NHB |
| Porifera | Demospongiae | Haplosclerida | Petrosiidae | *Petrosia* | *Petrosia ficiformis* | Megafauna | Benthic | Sessile | Filter feeder | Solitary | NHB |
| Porifera | Demospongiae | Haplosclerida | Chalinidae | *Haliclona* | *Haliclona fulva* | Megafauna | Benthic | Sessile | Filter feeder | Solitary | NHB |
| Porifera | Demospongiae | Haplosclerida | Chalinidae | *Haliclona* | sp. 1 | Megafauna | Benthic | Sessile | Filter feeder | Solitary | NHB |
| Porifera | Demospongiae | Haplosclerida | Chalinidae | *Haliclona* | sp. 2 | Megafauna | Benthic | Sessile | Filter feeder | Solitary | NHB |
| Porifera | Demospongiae | Haplosclerida | Chalinidae | *Haliclona* | sp. 3 | Megafauna | Benthic | Sessile | Filter feeder | Solitary | NHB |
| Porifera | Demospongiae | Haplosclerida | Chalinidae | *Haliclona* | sp. 4 | Megafauna | Benthic | Sessile | Filter feeder | Solitary | NHB |
| Porifera | Demospongiae | Haplosclerida | Chalinidae | *Haliclona* | sp. 5 | Megafauna | Benthic | Sessile | Filter feeder | Solitary | NHB |
| Porifera | Demospongiae | Haplosclerida | Chalinidae | *Haliclona* | sp. 6 | Megafauna | Benthic | Sessile | Filter feeder | Solitary | NHB |
| Porifera | Demospongiae | Haplosclerida | Chalinidae | *Haliclona* | sp. 7 | Megafauna | Benthic | Sessile | Filter feeder | Solitary | NHB |
| Porifera | Demospongiae | Haplosclerida | Chalinidae | *Haliclona* | sp. 8 | Megafauna | Benthic | Sessile | Filter feeder | Solitary | NHB |
| Porifera | Demospongiae | Haplosclerida | Chalinidae | *Haliclona* | sp. 9 | Megafauna | Benthic | Sessile | Filter feeder | Solitary | NHB |
| Porifera | Demospongiae | Haplosclerida | Chalinidae | *Haliclona* | sp. 10 | Megafauna | Benthic | Sessile | Filter feeder | Solitary | NHB |
| Porifera | Demospongiae | Haplosclerida | Chalinidae | *Haliclona* | sp. 11 | Megafauna | Benthic | Sessile | Filter feeder | Solitary | NHB |
| Porifera | Demospongiae | Haplosclerida | Chalinidae | *Haliclona* | sp. 12 | Megafauna | Benthic | Sessile | Filter feeder | Solitary | NHB |
| Porifera | Demospongiae | Poecilosclerida | Crambeidae | *Crambe* | *Crambe crambe* | Megafauna | Benthic | Sessile | Filter feeder | Solitary | NHB |
| Porifera | Demospongiae | Poecilosclerida | Crambeidae | *Crambe* | spp. | Megafauna | Benthic | Sessile | Filter feeder | Solitary | NHB |
| Porifera | Demospongiae | Poecilosclerida | Esperiopsidae | *Ulosa* | *Ulosa digitata* | Megafauna | Benthic | Sessile | Filter feeder | Solitary | NHB |
| Porifera | Demospongiae | Poecilosclerida | Hymedesmiidae | *Hemimycale* | *Hemimycale columella* | Megafauna | Benthic | Sessile | Filter feeder | Solitary | NHB |
| Porifera | Demospongiae | Poecilosclerida | Hymedesmiidae | *Phorbas* | *Phorbas tenacior* | Megafauna | Benthic | Sessile | Filter feeder | Solitary | NHB |
| Porifera | Demospongiae | Poecilosclerida | Microcionidae | *Clathria* | *Clathria compressa* | Megafauna | Benthic | Sessile | Filter feeder | Solitary | NHB |
| Porifera | Demospongiae | Poecilosclerida | Mycalidae | *Mycale* | sp. 1 | Megafauna | Benthic | Sessile | Filter feeder | Solitary | NHB |
| Porifera | Demospongiae | Poecilosclerida | Tedaniidae | *Tedania* | sp. 1 | Megafauna | Benthic | Sessile | Filter feeder | Solitary | NHB |
| Porifera | Demospongiae | Polymastiida | Polymastiidae | *Polymastia* | spp. | Megafauna | Benthic | Sessile | Filter feeder | Solitary | NHB |
| Porifera | Demospongiae | Suberitida | Halichondriidae | *Halichondria* | sp. 1 | Megafauna | Benthic | Sessile | Filter feeder | Solitary | NHB |
| Porifera | Demospongiae | Suberitida | Halichondriidae | *Halichondria* | sp. 2 | Megafauna | Benthic | Sessile | Filter feeder | Solitary | NHB |
| Porifera | Demospongiae | Suberitida | Halichondriidae | *Topsentia* | *Topsentia vaceleti* | Megafauna | Benthic | Sessile | Filter feeder | Solitary | NHB |
| Porifera | Demospongiae | Suberitida | Suberitidae | *Suberites* | spp. | Megafauna | Benthic | Sessile | Filter feeder | Solitary | NHB |
| Porifera | Demospongiae | Suberitida | Suberitidae | *Suberites* | *Suberites domuncula* | Megafauna | Benthic | Sessile | Filter feeder | Solitary | NHB |
| Porifera | Demospongiae | Tethyida | Tethyidae | *Tethya* | sp. 1 | Megafauna | Benthic | Sessile | Filter feeder | Solitary | NHB |
| Porifera | Demospongiae | Tethyida | Tethyidae | *Tethya* | *Tethya aurantium* | Megafauna | Benthic | Sessile | Filter feeder | Solitary | NHB |
| Porifera | Demospongiae | Tethyida | Tethyidae | *Tethya* | *Tethya citrina* | Megafauna | Benthic | Sessile | Filter feeder | Solitary | NHB |
| Porifera | Demospongiae | Tetractinellida | Geodiidae | *Geodia* | *Geodia cydonium* | Megafauna | Benthic | Sessile | Filter feeder | Solitary | NHB |
| Porifera | Demospongiae | Tetractinellida | Geodiidae | *Geodia* | sp. 1 | Megafauna | Benthic | Sessile | Filter feeder | Solitary | NHB |
| Porifera | Demospongiae | Tetractinellida | Pachastrellidae | *Pachastrella* | *Pachastrella monilifera* | Megafauna | Benthic | Sessile | Filter feeder | Solitary | HB |
| Porifera | Demospongiae | Tetractinellida | Theneidae | *Thenea* | *Thenea muricata* | Megafauna | Benthic | Sessile | Filter feeder | Solitary | NHB |
| Porifera | Demospongiae | Tetractinellida | Vulcanellidae | *Poecillastra* | *Poecillastra compressa* | Megafauna | Benthic | Sessile | Filter feeder | Solitary | HB |
| Porifera | Demospongiae | Verongiida | Aplysinidae | *Aplysina* | sp. 1 | Megafauna | Benthic | Sessile | Filter feeder | Solitary | NHB |
| Porifera | Demospongiae | Verongiida | Ianthellidae | *Hexadella* | *Hexadella racovitzai* | Megafauna | Benthic | Sessile | Filter feeder | Solitary | NHB |
| Porifera | Demospongiae | Verongiida | Ianthellidae | *Hexadella* | sp. 1 | Megafauna | Benthic | Sessile | Filter feeder | Solitary | NHB |
| Porifera | Demospongiae |  |  |  | sp. 1 | Megafauna | Benthic | Sessile | Filter feeder | Solitary | NHB |
| Porifera | Demospongiae |  |  |  | sp. 2 | Megafauna | Benthic | Sessile | Filter feeder | Solitary | NHB |
| Porifera | Demospongiae |  |  |  | sp. 3 | Megafauna | Benthic | Sessile | Filter feeder | Solitary | NHB |
| Porifera | Demospongiae |  |  |  | sp. 4 | Megafauna | Benthic | Sessile | Filter feeder | Solitary | NHB |
| Porifera | Demospongiae |  |  |  | sp. 5 | Megafauna | Benthic | Sessile | Filter feeder | Solitary | NHB |
| Porifera | Demospongiae |  |  |  | sp. 6 | Megafauna | Benthic | Sessile | Filter feeder | Solitary | NHB |
| Porifera | Demospongiae |  |  |  | sp. 7 | Megafauna | Benthic | Sessile | Filter feeder | Solitary | NHB |
| Porifera | Demospongiae |  |  |  | sp. 8 | Megafauna | Benthic | Sessile | Filter feeder | Solitary | NHB |
| Porifera | Demospongiae |  |  |  | sp. 9 | Megafauna | Benthic | Sessile | Filter feeder | Solitary | NHB |
| Porifera | Demospongiae |  |  |  | sp. 10 | Megafauna | Benthic | Sessile | Filter feeder | Solitary | NHB |
| Porifera | Demospongiae |  |  |  | sp. 11 | Megafauna | Benthic | Sessile | Filter feeder | Solitary | NHB |
| Porifera | Demospongiae |  |  |  | sp. 12 | Megafauna | Benthic | Sessile | Filter feeder | Solitary | NHB |
| Porifera | Demospongiae |  |  |  | sp. 13 | Megafauna | Benthic | Sessile | Filter feeder | Solitary | NHB |
| Porifera | Demospongiae |  |  |  | sp. 14 | Megafauna | Benthic | Sessile | Filter feeder | Solitary | NHB |
| Porifera | Demospongiae |  |  |  | sp. 15 | Megafauna | Benthic | Sessile | Filter feeder | Solitary | NHB |
| Porifera | Demospongiae |  |  |  | sp. 16 | Megafauna | Benthic | Sessile | Filter feeder | Solitary | NHB |
| Porifera | Demospongiae |  |  |  | sp. 17 | Megafauna | Benthic | Sessile | Filter feeder | Solitary | NHB |
| Porifera | Demospongiae |  |  |  | sp. 18 | Megafauna | Benthic | Sessile | Filter feeder | Solitary | NHB |
| Porifera | Demospongiae |  |  |  | sp. 19 | Megafauna | Benthic | Sessile | Filter feeder | Solitary | NHB |
| Porifera | Demospongiae |  |  |  | sp. 20 | Megafauna | Benthic | Sessile | Filter feeder | Solitary | NHB |
| Porifera | Demospongiae |  |  |  | sp. 21 | Megafauna | Benthic | Sessile | Filter feeder | Solitary | NHB |
| Porifera | Demospongiae |  |  |  | sp. 22 | Megafauna | Benthic | Sessile | Filter feeder | Solitary | NHB |
| Porifera | Demospongiae |  |  |  | sp. 23 | Megafauna | Benthic | Sessile | Filter feeder | Solitary | NHB |
| Porifera | Demospongiae |  |  |  | sp. 24 | Megafauna | Benthic | Sessile | Filter feeder | Solitary | NHB |
| Porifera | Demospongiae |  |  |  | sp. 25 | Megafauna | Benthic | Sessile | Filter feeder | Solitary | NHB |
| Porifera | Demospongiae |  |  |  | sp. 26 | Megafauna | Benthic | Sessile | Filter feeder | Solitary | NHB |
| Porifera | Demospongiae |  |  |  | sp. 27 | Megafauna | Benthic | Sessile | Filter feeder | Solitary | NHB |
| Porifera | Hexactinellida | Lyssacinosida | Rossellidae | *Sympagella* | *Sympagella delauzei* | Megafauna | Benthic | Sessile | Filter feeder | Solitary | NHB |
| Porifera | Homoscleromorpha | Homosclerophorida | Plakinidae | *Corticium* | spp. | Megafauna | Benthic | Sessile | Filter feeder | Solitary | NHB |
| Porifera | Homoscleromorpha | Homosclerophorida | Plakinidae | *Corticium* | *Corticium candelabrum* | Megafauna | Benthic | Sessile | Filter feeder | Solitary | NHB |
| Cnidaria | Anthozoa | Actiniaria |  |  | spp. | Megafauna | Benthic | Sessile | Suspension feeder | Solitary | NHB |
| Cnidaria | Anthozoa | Actiniaria | Aliciidae | *Alicia* | *Alicia mirabilis* | Megafauna | Benthic | Sessile | Suspension feeder | Solitary | NHB |
| Cnidaria | Anthozoa | Actiniaria | Andresiidae | *Andresia* | *Andresia partenopea* | Megafauna | Benthic | Sessile | Suspension feeder | Solitary | NHB |
| Cnidaria | Anthozoa | Alcyonacea | Acanthogorgiidae | *Acanthogorgia* | *Acanthogorgia hirsuta* | Megafauna | Benthic | Sessile | Suspension feeder | Colonial | NHB |
| Cnidaria | Anthozoa | Alcyonacea | Alcyoniidae | *Alcyonium* | *Alcyonium coralloides* | Megafauna | Benthic | Sessile | Suspension feeder | Colonial | NHB |
| Cnidaria | Anthozoa | Alcyonacea | Alcyoniidae | *Alcyonium* | *Alcyonium palmatum* | Megafauna | Benthic | Sessile | Suspension feeder | Colonial | NHB |
| Cnidaria | Anthozoa | Alcyonacea | Coralliidae | *Corallium* | *Corallium rubrum* | Megafauna | Benthic | Sessile | Suspension feeder | Colonial | HB |
| Cnidaria | Anthozoa | Alcyonacea | Cornulariidae | *Cornularia* | *Cornularia cornucopiae* | Macrofauna | Benthic | Sessile | Suspension feeder | Colonial | NHB |
| Cnidaria | Anthozoa | Alcyonacea | Gorgoniidae | *Eunicella* | spp. | Megafauna | Benthic | Sessile | Suspension feeder | Colonial | NHB |
| Cnidaria | Anthozoa | Alcyonacea | Gorgoniidae | *Eunicella* | *Eunicella cavolini* | Megafauna | Benthic | Sessile | Suspension feeder | Colonial | HB |
| Cnidaria | Anthozoa | Alcyonacea | Gorgoniidae | *Eunicella* | *Eunicella singularis* | Megafauna | Benthic | Sessile | Suspension feeder | Colonial | HB |
| Cnidaria | Anthozoa | Alcyonacea | Gorgoniidae | *Eunicella* | *Eunicella verrucosa* | Megafauna | Benthic | Sessile | Suspension feeder | Colonial | HB |
| Cnidaria | Anthozoa | Alcyonacea | Nidaliidae | *Nidalia* | *Nidalia studeri* | Megafauna | Benthic | Sessile | Suspension feeder | Colonial | NHB |
| Cnidaria | Anthozoa | Alcyonacea | Paralcyoniidae | *Paralcyonium* | *Paralcyonium spinulosum* | Megafauna | Benthic | Sessile | Suspension feeder | Colonial | NHB |
| Cnidaria | Anthozoa | Alcyonacea | Plexauridae | *Paramuricea* | *Paramuricea clavata* | Megafauna | Benthic | Sessile | Suspension feeder | Colonial | HB |
| Cnidaria | Anthozoa | Alcyonacea | Plexauridae | *Paramuricea* | *Paramuricea macrospina* | Megafauna | Benthic | Sessile | Suspension feeder | Colonial | HB |
| Cnidaria | Anthozoa | Alcyonacea | Plexauridae | *Swiftia* | *Swiftia pallida* | Megafauna | Benthic | Sessile | Filter feeder | Solitary | NHB |
| Cnidaria | Anthozoa | Alcyonacea | Primnoidae | *Callogorgia* | *Callogorgia verticillata* | Megafauna | Benthic | Sessile | Suspension feeder | Colonial | HB |
| Cnidaria | Anthozoa | Alcyonacea |  |  | sp. 1 | Megafauna | Benthic | Sessile | Suspension feeder | Colonial | NHB |
| Cnidaria | Anthozoa | Antipatharia | Myriopathidae | *Antipathella* | *Antipathella subpinnata* | Megafauna | Benthic | Sessile | Suspension feeder | Colonial | HB |
| Cnidaria | Anthozoa | Penicillaria | Arachnactidae | *Arachnanthus* | *Arachnanthus oligopodus* | Megafauna | Benthic | Sessile | Suspension feeder | Solitary | NHB |
| Cnidaria | Anthozoa | Pennatulacea | Funiculinidae | *Funiculina* | *Funiculina quadrangularis* | Megafauna | Benthic | Sessile | Suspension feeder | Colonial | HB |
| Cnidaria | Anthozoa | Pennatulacea | Pennatulidae | *Pennatula* | *Pennatula phosphorea* | Megafauna | Benthic | Sessile | Suspension feeder | Colonial | HB |
| Cnidaria | Anthozoa | Pennatulacea | Pennatulidae | *Pennatula* | *Pennatula rubra* | Megafauna | Benthic | Sessile | Suspension feeder | Colonial | HB |
| Cnidaria | Anthozoa | Pennatulacea | Virgulariidae | *Virgularia* | *Virgularia mirabilis* | Megafauna | Benthic | Sessile | Suspension feeder | Colonial | HB |
| Cnidaria | Anthozoa | Scleractinia | Caryophylliidae |  | spp. | Megafauna | Benthic | Sessile | Suspension feeder | Solitary | HB |
| Cnidaria | Anthozoa | Scleractinia | Caryophylliidae | *Caryophyllia* | sp. 1 | Megafauna | Benthic | Sessile | Suspension feeder | Solitary | HB |
| Cnidaria | Anthozoa | Scleractinia | Caryophylliidae | *Caryophyllia* | sp. 2 | Megafauna | Benthic | Sessile | Suspension feeder | Solitary | HB |
| Cnidaria | Anthozoa | Scleractinia | Caryophylliidae |  | sp. 1 | Megafauna | Benthic | Sessile | Suspension feeder | Solitary | HB |
| Cnidaria | Anthozoa | Scleractinia | Caryophylliidae |  | sp. 2 | Megafauna | Benthic | Sessile | Suspension feeder | Solitary | HB |
| Cnidaria | Anthozoa | Scleractinia | Dendrophylliidae | *Balanophyllia* | *Balanophyllia europaea* | Megafauna | Benthic | Sessile | Suspension feeder | Solitary | HB |
| Cnidaria | Anthozoa | Scleractinia | Dendrophylliidae | *Balanophyllia* | sp. 1 | Megafauna | Benthic | Sessile | Suspension feeder | Solitary | HB |
| Cnidaria | Anthozoa | Scleractinia | Dendrophylliidae | *Dendrophyllia* | *Dendrophyllia cornigera* | Megafauna | Benthic | Sessile | Suspension feeder | Colonial | HB |
| Cnidaria | Anthozoa | Spirularia | Cerianthidae | *Cerianthus* | *Cerianthus membranaceus* | Megafauna | Benthic | Sessile | Scavenger/Predator | Solitary | NHB |
| Cnidaria | Anthozoa | Spirularia | Cerianthidae |  | spp. | Megafauna | Benthic | Sessile | Scavenger/Predator | Solitary | NHB |
| Cnidaria | Anthozoa | Spirularia | Cerianthidae | *Cerianthus* | spp. | Megafauna | Benthic | Sessile | Scavenger/Predator | Solitary | NHB |
| Cnidaria | Anthozoa | Spirularia | Cerianthidae |  | sp. 1 | Megafauna | Benthic | Sessile | Scavenger/Predator | Solitary | NHB |
| Cnidaria | Anthozoa | Zoantharia | Epizoanthidae | *Epizoanthus* | sp. 1 | Megafauna | Benthic | Sessile | Suspension feeder | Solitary | NHB |
| Cnidaria | Anthozoa | Zoantharia | Epizoanthidae | *Epizoanthus* | sp. 2 | Megafauna | Benthic | Sessile | Suspension feeder | Solitary | NHB |
| Cnidaria | Anthozoa | Zoantharia | Parazoanthidae | *Parazoanthus* | sp. 1 | Megafauna | Benthic | Sessile | Suspension feeder | Solitary | NHB |
| Cnidaria | Hydrozoa | Leptothecata | Aglaopheniidae | *Aglaophenia* | sp. 1 | Megafauna | Benthic | Sessile | Suspension feeder | Colonial | NHB |
| Cnidaria | Hydrozoa | Leptothecata | Aglaopheniidae | *Lytocarpia* | *Lytocarpia myriophyllum* | Megafauna | Benthic | Sessile | Suspension feeder | Colonial | HB |
| Cnidaria | Hydrozoa | Leptothecata | Plumulariidae | *Nemertesia* | spp. | Megafauna | Benthic | Sessile | Suspension feeder | Colonial | NHB |
| Mollusca | Bivalvia | Cardiida | Cardiidae | *Acanthocardia* | *Acanthocardia Aculeata* | Megafauna | Benthic | Sessile | Filter feeder | Solitary | NHB |
| Mollusca | Bivalvia | Cardiida | Cardiidae | *Acanthocardia* | sp. 1 | Megafauna | Benthic | Sessile | Filter feeder | Solitary | NHB |
| Mollusca | Bivalvia | Ostreida | Gryphaeidae | *Neopycnodonte* | *Neopycnodonte cochlear* | Megafauna | Benthic | Sessile | Suspension feeder | Solitary | HB |
| Mollusca | Bivalvia | Ostreida | Pteriidae | *Pteria* | *Pteria hirundo* | Megafauna | Benthic | Sessile | Suspension feeder | Solitary | NHB |
| Mollusca | Bivalvia | Pectinida | Pectinidae | *Pecten* | spp. | Megafauna | Benthic | Sessile | Suspension feeder | Solitary | HB |
| Mollusca | Cephalopoda | Octopoda | Eledonidae | *Eledone* | *Eledone cirrhosa* | Megafauna | Benthic | Swimmer | Scavenger/Predator | Solitary | NHB |
| Mollusca | Cephalopoda | Octopoda | Octopodidae | *Callistoctopus* | *Callistoctopus macropus* | Megafauna | Benthic | Swimmer | Scavenger/Predator | Solitary | NHB |
| Mollusca | Cephalopoda | Octopoda | Octopodidae | *Octopus* | *Octopus vulgaris* | Megafauna | Benthic | Swimmer | Scavenger/Predator | Solitary | NHB |
| Mollusca | Cephalopoda | Sepiida | Sepiidae | *Sepia* | spp. | Megafauna | Benthic | Swimmer | Scavenger/Predator | Solitary | NHB |
| Mollusca | Gastropoda |  | Plakobranchidae | *Elysia* | sp. 1 | Megafauna | Benthic | Vagile | Grazer | Solitary | NHB |
| Mollusca | Gastropoda |  |  |  | spp. | Megafauna | Benthic | Vagile | Scavenger/Predator | Solitary | NHB |
| Mollusca | Gastropoda |  |  |  | sp. 1 | Megafauna | Benthic | Vagile | Scavenger/Predator | Solitary | NHB |
| Mollusca | Gastropoda |  |  |  | sp. 2 | Megafauna | Benthic | Vagile | Scavenger/Predator | Solitary | NHB |
| Mollusca | Gastropoda | Littorinimorpha | Cypraeidae | *Naria* | spp. | Megafauna | Benthic | Vagile | Scavenger/Predator | Solitary | NHB |
| Mollusca | Gastropoda | Neogastropoda | Columbellidae | *Columbella* | *Columbella rustica* | Macrofauna | Benthic | Vagile | Grazer | Solitary | NHB |
| Mollusca | Gastropoda | Neogastropoda | Fasciolariidae | *Fusinus* | spp. | Megafauna | Benthic | Vagile | Scavenger/Predator | Solitary | NHB |
| Mollusca | Gastropoda | Neogastropoda | Fasciolariidae | *Tarantinaea* | spp. | Megafauna | Benthic | Vagile | Scavenger/Predator | Solitary | NHB |
| Mollusca | Gastropoda | Neogastropoda | Muricidae | *Hexaplex* | *Hexaplex trunculus* | Megafauna | Benthic | Vagile | Scavenger/Predator | Solitary | NHB |
| Mollusca | Gastropoda | Nudibranchia | Chromodorididae | *Felimare* | *Felimare tricolor* | Megafauna | Benthic | Vagile | Scavenger/Predator | Solitary | NHB |
| Mollusca | Gastropoda | Nudibranchia | Discodorididae | *Discodoris* | spp. | Megafauna | Benthic | Vagile | Scavenger/Predator | Solitary | NHB |
| Mollusca | Gastropoda | Nudibranchia | Discodorididae | *Platydoris* | *Platydoris argo* | Megafauna | Benthic | Vagile | Scavenger/Predator | Solitary | NHB |
| Mollusca | Gastropoda | Nudibranchia | Myrrhinidae | *Dondice* | *Dondice banyulensis* | Megafauna | Benthic | Vagile | Scavenger/Predator | Solitary | NHB |
| Mollusca | Gastropoda | Pleurobranchida | Pleurobranchidae | *Pleurobranchus* | *Pleurobranchus testudinarius* | Megafauna | Benthic | Vagile | Scavenger/Predator | Solitary | NHB |
| Mollusca | Gastropoda | Trochida | Calliostomatidae | *Calliostoma* | spp. | Macrofauna | Benthic | Vagile | Scavenger/Predator | Solitary | NHB |
| Mollusca | Gastropoda | Trochida | Turbinidae | *Bolma* | *Bolma rugosa* | Megafauna | Benthic | Vagile | Grazer | Solitary | NHB |
|  |  |  |  |  |  |  |  |  |  |  |  |
|  |  |  |  |  |  |  |  |  |  |  |  |
|  |  |  |  |  |  |  |  |  |  |  |  |
|  |  |  |  |  |  |  |  |  |  |  |  |
| Annelida | Polychaeta | Echiuroidea | Bonelliidae | *Bonellia* | *Bonellia viridis* | Megafauna | Benthic | Vagile | Deposit feeder | Solitary | NHB |
| Annelida | Polychaeta | Sabellida | Sabellidae |  | spp. | Megafauna | Benthic | Sessile | Suspension feeder | Solitary | NHB |
| Annelida | Polychaeta | Sabellida | Sabellidae |  | sp. 1 | Megafauna | Benthic | Sessile | Suspension feeder | Solitary | NHB |
| Annelida | Polychaeta | Sabellida | Sabellidae | *Sabella* | spp. | Megafauna | Benthic | Sessile | Suspension feeder | Solitary | NHB |
| Annelida | Polychaeta | Sabellida | Sabellidae | *Sabella* | sp. 1 | Megafauna | Benthic | Sessile | Suspension feeder | Solitary | NHB |
| Annelida | Polychaeta | Sabellida | Sabellidae | *Sabella* | *Sabella pavonina* | Megafauna | Benthic | Sessile | Suspension feeder | Solitary | NHB |
| Annelida | Polychaeta | Sabellida | Serpulidae |  | spp. | Megafauna | Benthic | Sessile | Suspension feeder | Solitary | NHB |
| Annelida | Polychaeta | Sabellida | Serpulidae | *Filograna* | *Filograna implexa* | Macrofauna | Benthic | Sessile | Filter feeder | Gregarious | HB |
| Annelida | Polychaeta | Sabellida | Serpulidae | *Protula* | sp. 1 | Megafauna | Benthic | Sessile | Filter feeder | Solitary | NHB |
| Annelida | Polychaeta | Sabellida | Serpulidae | *Serpula* | *Serpula vermicularis* | Megafauna | Benthic | Sessile | Suspension feeder | Solitary | NHB |
| Annelida | Polychaeta | Sabellida | Serpulidae | *Vermiliopsis* | sp. 1 | Megafauna | Benthic | Sessile | Filter feeder | Solitary | NHB |
| Annelida | Polychaeta | Terebellida | Terebellidae | *Lanice* | *Lanice conchilega* | Megafauna | Benthic | Facultatively motile | Suspension feeder | Solitary | NHB |
| Annelida | Polychaeta | Terebellida | Trichobranchidae | *Terebellides* | sp. 1 | Megafauna | Benthic | Facultatively motile | Deposit feeder | Solitary | NHB |
| Arthropoda | Malacostraca | Decapoda | Calappidae | *Calappa* | *Calappa granulata* | Megafauna | Benthic | Vagile | Scavenger/Predator | Solitary | NHB |
| Arthropoda | Malacostraca | Decapoda | Inachidae | *Inachus* | *Inachus phalangium* | Megafauna | Benthic | Vagile | Scavenger/Predator | Solitary | NHB |
| Arthropoda | Malacostraca | Decapoda | Inachidae | *Macropodia* | *Macropodia longirostris* | Megafauna | Benthic | Vagile | Scavenger/Predator | Solitary | NHB |
| Arthropoda | Malacostraca | Decapoda | Munididae | *Munida* | spp. | Megafauna | Benthic | Vagile | Scavenger/Predator | Solitary | NHB |
| Arthropoda | Malacostraca | Decapoda | Munididae | *Munida* | *Munida tenuimana* | Megafauna | Benthic | Vagile | Scavenger/Predator | Solitary | NHB |
| Arthropoda | Malacostraca | Decapoda | Paguridae | *Paguristes* | *Paguristes longirostris* | Megafauna | Benthic | Vagile | Scavenger/Predator | Solitary | NHB |
| Arthropoda | Malacostraca | Decapoda | Paguridae |  | spp. | Megafauna | Benthic | Vagile | Scavenger/Predator | Solitary | NHB |
| Arthropoda | Malacostraca | Decapoda | Paguridae |  | sp. 1 | Megafauna | Benthic | Vagile | Scavenger/Predator | Solitary | NHB |
| Arthropoda | Malacostraca | Decapoda | Paguridae |  | sp. 2 | Megafauna | Benthic | Vagile | Scavenger/Predator | Solitary | NHB |
| Arthropoda | Malacostraca | Decapoda | Paguridae |  | sp. 3 | Megafauna | Benthic | Vagile | Scavenger/Predator | Solitary | NHB |
| Arthropoda | Malacostraca | Decapoda | Paguridae |  | sp. 4 | Megafauna | Benthic | Vagile | Scavenger/Predator | Solitary | NHB |
| Arthropoda | Malacostraca | Decapoda | Palinuridae | *Palinurus* | *Palinurus elephas* | Megafauna | Benthic | Vagile | Scavenger/Predator | Solitary | NHB |
| Arthropoda | Malacostraca | Decapoda | Parthenopidae | *Spinolambrus* | spp. | Megafauna | Benthic | Vagile | Scavenger/Predator | Solitary | NHB |
| Arthropoda | Malacostraca | Decapoda | Polybiidae | *Liocarcinus* | *Liocarcinus depurator* | Megafauna | Benthic | Vagile | Scavenger/Predator | Solitary | NHB |
| Arthropoda | Malacostraca | Decapoda | Polybiidae | *Liocarcinus* | spp. | Megafauna | Benthic | Vagile | Scavenger/Predator | Solitary | NHB |
| Arthropoda | Malacostraca | Decapoda |  |  | spp. | Megafauna | Benthic | Vagile | Scavenger/Predator | Solitary | NHB |
| Arthropoda | Malacostraca | Decapoda |  |  | sp. 1 | Megafauna | Benthic | Vagile | Scavenger/Predator | Solitary | NHB |
| Arthropoda | Malacostraca | Decapoda |  |  | sp. 2 | Megafauna | Benthic | Vagile | Scavenger/Predator | Solitary | NHB |
| Arthropoda | Malacostraca | Euphausiacea |  |  | spp. | Megafauna | Pelagic | Vagile | Scavenger/Predator | Solitary | NHB |
| Bryozoa | Gymnolaemata | Cheilostomatida | Adeonidae | *Adeonella* | *Adeonella calveti* | Megafauna | Benthic | Sessile | Suspension feeder | Colonial | NHB |
| Bryozoa | Gymnolaemata | Cheilostomatida | Bitectiporidae | *Pentapora* | *Pentapora fascialis* | Megafauna | Benthic | Sessile | Suspension feeder | Colonial | NHB |
| Bryozoa | Gymnolaemata | Cheilostomatida | Bitectiporidae | *Schizomavella* | *Schizomavella linearis* | Megafauna | Benthic | Sessile | Suspension feeder | Colonial | NHB |
| Bryozoa | Gymnolaemata | Cheilostomatida | Bitectiporidae | *Schizomavella* | *Schizomavella mamillata* | Megafauna | Benthic | Sessile | Suspension feeder | Colonial | NHB |
| Bryozoa | Gymnolaemata | Cheilostomatida | Bitectiporidae | *Schizomavella* | sp. 1 | Megafauna | Benthic | Sessile | Suspension feeder | Colonial | NHB |
| Bryozoa | Gymnolaemata | Cheilostomatida | Bugulidae | *Bugula* | spp. | Megafauna | Benthic | Sessile | Suspension feeder | Colonial | NHB |
| Bryozoa | Gymnolaemata | Cheilostomatida | Celleporidae | *Turbicellepora* | *Turbicellepora avicularis* | Megafauna | Benthic | Sessile | Suspension feeder | Colonial | NHB |
| Bryozoa | Gymnolaemata | Cheilostomatida | Myriaporidae | *Myriapora* | *Myriapora truncata* | Megafauna | Benthic | Sessile | Suspension feeder | Colonial | NHB |
| Bryozoa | Gymnolaemata | Cheilostomatida | Myriaporidae | *Myriapora* | spp. | Megafauna | Benthic | Sessile | Suspension feeder | Colonial | NHB |
| Bryozoa | Gymnolaemata | Cheilostomatida | Phidoloporidae | *Reteporella* | *Reteporella grimaldii* | Megafauna | Benthic | Sessile | Suspension feeder | Solitary | NHB |
| Bryozoa | Gymnolaemata | Cheilostomatida | Phidoloporidae | *Reteporella* | spp. | Megafauna | Benthic | Sessile | Suspension feeder | Solitary | NHB |
| Bryozoa | Gymnolaemata | Cheilostomatida | Phidoloporidae | *Reteporella* | sp. 1 | Megafauna | Benthic | Sessile | Suspension feeder | Solitary | NHB |
| Bryozoa | Gymnolaemata | Cheilostomatida | Smittinidae | *Smittina* | *Smittina cervicornis* | Megafauna | Benthic | Sessile | Suspension feeder | Colonial | NHB |
| Bryozoa | Stenolaemata | Cyclostomatida | Horneridae | *Hornera* | *Hornera frondiculata* | Megafauna | Benthic | Sessile | Suspension feeder | Colonial | NHB |
| Echinodermata | Asteroidea | Forcipulatida | Asteriidae | *Coscinasterias* | *Coscinasterias tenuispina* | Megafauna | Benthic | Sessile | Scavenger/Predator | Solitary | NHB |
| Echinodermata | Asteroidea | Forcipulatida | Asteriidae | *Marthasterias* | *Marthasterias glacialis* | Megafauna | Benthic | Sessile | Scavenger/Predator | Solitary | NHB |
| Echinodermata | Asteroidea | Paxillosida | Astropectinidae | *Astropecten* | spp. | Megafauna | Benthic | Sessile | Scavenger/Predator | Solitary | NHB |
| Echinodermata | Asteroidea | Paxillosida | Astropectinidae | *Astropecten* | *Astropecten aranciacus* | Megafauna | Benthic | Sessile | Scavenger/Predator | Solitary | NHB |
| Echinodermata | Asteroidea | Paxillosida | Luidiidae | *Luidia* | *Luidia ciliaris* | Megafauna | Benthic | Sessile | Scavenger/Predator | Solitary | NHB |
| Echinodermata | Asteroidea | Spinulosida | Echinasteridae | *Echinaster* | *Echinaster sepositus* | Megafauna | Benthic | Sessile | Scavenger/Predator | Solitary | NHB |
| Echinodermata | Asteroidea | Valvatida | Chaetasteridae | *Chaetaster* | *Chaetaster longipes* | Megafauna | Benthic | Sessile | Scavenger/Predator | Solitary | NHB |
| Echinodermata | Asteroidea | Valvatida | Goniasteridae | *Peltaster* | *Peltaster placenta* | Megafauna | Benthic | Sessile | Deposit feeder | Solitary | NHB |
| Echinodermata | Asteroidea | Valvatida | Ophidiasteridae | *Hacelia* | *Hacelia attenuata* | Megafauna | Benthic | Sessile | Deposit feeder | Solitary | NHB |
| Echinodermata | Crinoidea | Comatulida | Antedonidae | *Antedon* | *Antedon mediterranea* | Megafauna | Benthic | Facultatively motile | Suspension feeder | Solitary | NHB |
| Echinodermata | Crinoidea | Comatulida | Antedonidae | *Leptometra* | *Leptometra phalangium* | Megafauna | Benthic | Facultatively motile | Suspension feeder | Solitary | NHB |
| Echinodermata | Echinoidea | Camarodonta | Echinidae | *Echinus* | *Echinus melo* | Megafauna | Benthic | Vagile | Grazer | Solitary | NHB |
| Echinodermata | Echinoidea | Cidaroida | Cidaridae | *Cidaris* | *Stylocidaris affinis* | Megafauna | Benthic | Vagile | Scavenger/Predator | Solitary | NHB |
| Echinodermata | Echinoidea | Cidaroida | Cidaridae | *Cidaris* | *Cidaris cidaris* | Megafauna | Benthic | Vagile | Grazer | Solitary | NHB |
| Echinodermata | Echinoidea | Diadematoida | Diadematidae | *Centrostephanus* | *Centrostephanus longispinus* | Megafauna | Benthic | Vagile | Grazer | Solitary | NHB |
| Echinodermata | Echinoidea | Holothuriida | Holothuriidae | *Holothuria* | *Holothuria forskali* | Megafauna | Benthic | Vagile | Deposit feeder | Solitary | NHB |
| Echinodermata | Echinoidea | Holothuriida | Holothuriidae | *Holothuria* | *Holothuria poli* | Megafauna | Benthic | Vagile | Deposit feeder | Solitary | NHB |
| Echinodermata | Echinoidea | Holothuriida | Holothuriidae | *Holothuria* | spp. | Megafauna | Benthic | Vagile | Deposit feeder | Solitary | NHB |
| Echinodermata | Echinoidea | Holothuriida | Holothuriidae | *Holothuria* | *Holothuria tubulosa* | Megafauna | Benthic | Vagile | Deposit feeder | Solitary | NHB |
| Echinodermata | Echinoidea | Spatangoida | Brissidae | *Brissus* | *Brissus unicolor* | Megafauna | Benthic | Vagile | Deposit feeder | Solitary | NHB |
| Echinodermata | Echinoidea | Spatangoida | Spatangidae | *Spatangus* | *Spatangus purpureus* | Megafauna | Benthic | Vagile | Deposit feeder | Solitary | NHB |
| Echinodermata | Holothuroidea | Dendrochirotida | Cucumariidae | *Cucumaria* | *Cucumaria piperata* | Megafauna | Benthic | Vagile | Deposit feeder | Solitary | NHB |
| Echinodermata | Holothuroidea | Holothuriida | Mesothuriidae | *Mesothuria* | spp. | Megafauna | Benthic | Vagile | Deposit feeder | Solitary | NHB |
| Echinodermata | Holothuroidea | Synallactida | Stichopodidae | *Parastichopus* | *Parastichopus regalis* | Megafauna | Benthic | Vagile | Deposit feeder | Solitary | NHB |
| Echinodermata | Ophiuroidea |  |  |  | sp. 1 | Megafauna | Benthic | Vagile | Scavenger/Predator | Solitary | NHB |
| Echinodermata | Ophiuroidea | Amphilepidida | Ophiopsilidae | *Ophiopsila* | spp. | Megafauna | Benthic | Vagile | Suspension feeder | Solitary | NHB |
| Echinodermata | Ophiuroidea | Amphilepidida | Ophiotrichidae | *Ophiothrix* | *Ophiothrix fragilis* | Megafauna | Benthic | Vagile | Scavenger/Predator | Solitary | NHB |
| Echinodermata | Ophiuroidea | Euryalida | Gorgonocephalidae | *Astrospartus* | *Astrospartus mediterraneus* | Megafauna | Benthic | Vagile | Suspension feeder | Solitary | NHB |
| Echinodermata | Ophiuroidea | Ophiacanthida | Ophiodermatidae | *Ophioderma* | spp. | Megafauna | Benthic | Vagile | Scavenger/Predator | Solitary | NHB |
| Echinodermata | Ophiuroidea | Ophiacanthida | Ophiomyxidae | *Ophiomyxa* | *Ophiomyxa pentagona* | Megafauna | Benthic | Vagile | Scavenger/Predator | Solitary | NHB |
| Chordata | Actinopterygii | Anguilliformes |  |  | spp. | Megafauna | Pelagic | Swimmer | Scavenger/Predator | Solitary | NHB |
| Chordata | Actinopterygii |  |  |  | sp. 1 | Megafauna | Pelagic | Swimmer | Scavenger/Predator | Solitary | NHB |
| Chordata | Actinopterygii | Anguilliformes | Congridae | *Ariosoma* | *Ariosoma balearicum* | Megafauna | Pelagic | Swimmer | Scavenger/Predator | Solitary | NHB |
| Chordata | Actinopterygii | Anguilliformes | Muraenidae | *Muraena* | *Muraena helena* | Megafauna | Pelagic | Swimmer | Scavenger/Predator | Solitary | NHB |
| Chordata | Actinopterygii | Anguilliformes | Ophichthidae | *Ophisurus* | *Ophisurus serpens* | Megafauna | Pelagic | Swimmer | Scavenger/Predator | Solitary | NHB |
| Chordata | Actinopterygii | Gadiformes | Phycidae | *Phycis* | *Phycis blennoides* | Megafauna | Pelagic | Swimmer | Scavenger/Predator | Solitary | NHB |
| Chordata | Actinopterygii | Gadiformes | Phycidae | *Phycis* | spp. | Megafauna | Pelagic | Swimmer | Scavenger/Predator | Solitary | NHB |
| Chordata | Actinopterygii | Gadiformes | Phycidae | *Phycis* | *Phycis phycis* | Megafauna | Pelagic | Swimmer | Scavenger/Predator | Solitary | NHB |
| Chordata | Actinopterygii | Perciformes | Callanthiidae | *Callanthias* | *Callanthias ruber* | Megafauna | Pelagic | Swimmer | Scavenger/Predator | Solitary | NHB |
| Chordata | Actinopterygii | Perciformes | Gobiidae |  | spp. | Megafauna | Pelagic | Swimmer | Scavenger/Predator | Solitary | NHB |
| Chordata | Actinopterygii | Perciformes | Labridae | *Ctenolabrus* | *Ctenolabrus rupestris* | Megafauna | Pelagic | Swimmer | Scavenger/Predator | Solitary | NHB |
| Chordata | Actinopterygii | Perciformes | Labridae | *Lappanella* | *Lappanella fasciata* | Megafauna | Pelagic | Swimmer | Scavenger/Predator | Solitary | NHB |
| Chordata | Actinopterygii | Perciformes | Mullidae | *Mullus* | spp. | Megafauna | Pelagic | Swimmer | Scavenger/Predator | Solitary | NHB |
| Chordata | Actinopterygii | Perciformes | Mullidae | *Mullus* | *Mullus barbatus* | Megafauna | Pelagic | Swimmer | Scavenger/Predator | Solitary | NHB |
| Chordata | Actinopterygii | Perciformes | Pomacentridae | *Chromis* | spp. | Megafauna | Pelagic | Swimmer | Scavenger/Predator | Solitary | NHB |
| Chordata | Actinopterygii | Perciformes | Scorpaenidae | *Scorpaena* | *Scorpaena scrofa* | Megafauna | Pelagic | Swimmer | Scavenger/Predator | Solitary | NHB |
| Chordata | Actinopterygii | Perciformes | Serranidae | *Serranus* | spp. | Megafauna | Pelagic | Swimmer | Scavenger/Predator | Solitary | NHB |
| Chordata | Actinopterygii | Perciformes | Serranidae | *Serranus* | *Serranus cabrilla* | Megafauna | Pelagic | Swimmer | Scavenger/Predator | Solitary | NHB |
| Chordata | Actinopterygii | Perciformes | Serranidae | *Serranus* | *Serranus hepatus* | Megafauna | Pelagic | Swimmer | Scavenger/Predator | Solitary | NHB |
| Chordata | Actinopterygii | Perciformes | Serranidae |  | sp. 1 | Megafauna | Pelagic | Swimmer | Scavenger/Predator | Solitary | NHB |
| Chordata | Actinopterygii | Perciformes | Sparidae |  | spp. | Megafauna | Pelagic | Swimmer | Scavenger/Predator | Solitary | NHB |
| Chordata | Actinopterygii | Perciformes | Sparidae | *Pagellus* | spp. | Megafauna | Pelagic | Swimmer | Scavenger/Predator | Solitary | NHB |
| Chordata | Actinopterygii | Perciformes | Sparidae | *Pagellus* | *Pagellus erythrinus* | Megafauna | Pelagic | Swimmer | Scavenger/Predator | Solitary | NHB |
| Chordata | Actinopterygii | Perciformes | Sparidae | *Spondyliosoma* | *Spondyliosoma cantharus* | Megafauna | Pelagic | Swimmer | Scavenger/Predator | Solitary | NHB |
| Chordata | Actinopterygii | Perciformes | Synodontidae | *Synodus* | *Synodus saurus* | Megafauna | Pelagic | Swimmer | Scavenger/Predator | Solitary | NHB |
| Chordata | Actinopterygii | Perciformes | Trachinidae | *Trachinus* | spp. | Megafauna | Pelagic | Swimmer | Scavenger/Predator | Solitary | NHB |
| Chordata | Actinopterygii | Perciformes | Trachinidae | *Trachinus* | *Trachinus araneus* | Megafauna | Pelagic | Swimmer | Scavenger/Predator | Solitary | NHB |
| Chordata | Actinopterygii | Perciformes | Triglidae | *Eutrigla* | *Eutrigla* | Megafauna | Pelagic | Swimmer | Scavenger/Predator | Solitary | NHB |
| Chordata | Actinopterygii | Pleuronectiformes | Soleidae | *Solea* | *Solea solea* | Megafauna | Pelagic | Swimmer | Scavenger/Predator | Solitary | NHB |
| Chordata | Actinopterygii | Scorpaeniformes | Sebastidae | *Helicolenus* | *Helicolenus dactylopterus* | Megafauna | Benthic | Swimmer | Scavenger/Predator | Solitary | NHB |
| Chordata | Actinopterygii | Scorpaeniformes | Triglidae | *Chelidonichthys* | *Chelidonichthys lastoviza* | Megafauna | Benthic | Swimmer | Scavenger/Predator | Solitary | NHB |
| Chordata | Actinopterygii | Scorpaeniformes | Triglidae | *Chelidonichthys* | *Chelidonichthys lucerna* | Megafauna | Benthic | Swimmer | Scavenger/Predator | Solitary | NHB |
| Chordata | Actinopterygii | Scorpaeniformes | Triglidae | *Lepidotrigla* | *Lepidotrigla cavillone* | Megafauna | Benthic | Swimmer | Scavenger/Predator | Solitary | NHB |
| Chordata | Actinopterygii | Zeiformes | Zeidae | *Zeus* | *Zeus faber* | Megafauna | Pelagic | Swimmer | Scavenger/Predator | Solitary | NHB |
| Chordata | Ascidiacea | Aplousobranchia | Clavelinidae | *Clavelina* | sp. 1 | Megafauna | Benthic | Sessile | Filter feeder | Solitary | NHB |
| Chordata | Ascidiacea | Aplousobranchia | Didemnidae |  | sp. 1 | Megafauna | Benthic | Sessile | Filter feeder | Colonial | NHB |
| Chordata | Ascidiacea | Aplousobranchia | Didemnidae |  | sp. 2 | Megafauna | Benthic | Sessile | Filter feeder | Colonial | NHB |
| Chordata | Ascidiacea | Aplousobranchia | Didemnidae |  | sp. 3 | Megafauna | Benthic | Sessile | Filter feeder | Colonial | NHB |
| Chordata | Ascidiacea | Aplousobranchia | Diazonidae | *Rhopalaea* | spp. | Megafauna | Benthic | Sessile | Filter feeder | Solitary | NHB |
| Chordata | Ascidiacea | Aplousobranchia | Diazonidae | *Rhopalaea* | *Rhopalaea neapolitana* | Megafauna | Benthic | Sessile | Filter feeder | Solitary | NHB |
| Chordata | Ascidiacea | Aplousobranchia | Didemnidae | *Diplosoma* | *Diplosoma spongiforme* | Megafauna | Benthic | Sessile | Filter feeder | Colonial | NHB |
| Chordata | Ascidiacea | Aplousobranchia | Didemnidae | *Polysyncraton* | *Polysyncraton lacazei* | Megafauna | Benthic | Sessile | Filter feeder | Colonial | NHB |
| Chordata | Ascidiacea | Aplousobranchia | Polyclinidae | *Aplidium* | sp. 1 | Megafauna | Benthic | Sessile | Filter feeder | Colonial | NHB |
| Chordata | Ascidiacea | Phlebobranchia | Cionidae | *Ciona* | spp. | Megafauna | Benthic | Sessile | Filter feeder | Solitary | NHB |
| Chordata | Ascidiacea | Stolidobranchia | Pyuridae | *Halocynthia* | *Halocynthia papillosa* | Megafauna | Benthic | Sessile | Filter feeder | Solitary | NHB |
| Chordata | Ascidiacea |  |  |  | spp. | Megafauna | Benthic | Sessile | Filter feeder | Solitary | NHB |
| Chordata | Ascidiacea |  |  |  | sp. 1 | Megafauna | Benthic | Sessile | Filter feeder | Solitary | NHB |
| Chordata | Ascidiacea |  |  |  | sp. 2 | Megafauna | Benthic | Sessile | Filter feeder | Solitary | NHB |
| Chordata | Ascidiacea |  |  |  | sp. 3 | Megafauna | Benthic | Sessile | Filter feeder | Solitary | NHB |
| Chordata | Ascidiacea |  |  |  | sp. 4 | Megafauna | Benthic | Sessile | Filter feeder | Solitary | NHB |

**Tab. S3.** Results of the SIMPER analysis indicating the percentage of dissimilarity between groups identified with the hierarchical clustering explained by the abundance of Functional Entities.

| Depth  Range | Contrast | Functional entity | Contribution (%) | Cumulative contribution (%) |
| --- | --- | --- | --- | --- |
| All | **C+CN+DWOR - RB+SB** | Megafauna; Benthic; Sessile; Filter Feeders; Solitary; Non-habitat builders | 40.78 | 40.78 |
| All | **C+CN+DWOR - RB+SB** | Megafauna; Benthic; Sessile; Suspension Feeders; Colonial; Non-habitat builders | 22.37 | 63.15 |
| All | **C+CN+DWOR - RB+SB** | Megafauna; Benthic; Sessile; Suspension Feeders; Solitary; Habitat builders | 10.08 | 73.23 |
| 55-100 m | **C+DWOR - RB+SB+DWOR** | Megafauna; Benthic; Sessile; Suspension Feeders; Colonial; Non-habitat builders | 28.968 | 28.968 |
| 55-100 m | **C+DWOR - RB+SB+DWOR** | Megafauna; Benthic; Sessile; Filter Feeders; Solitary; Non-habitat builders | 26.074 | 55.042 |
| 55-100 m | **C+DWOR - RB+SB+DWOR** | Megafauna; Benthic; Sessile; Suspension Feeders; Solitary; Habitat builders | 11.251 | 66.293 |
| 55-100 m | **C+DWOR - RB+SB+DWOR** | Megafauna; Benthic; Sessile; Scavenger/Predator; Solitary; Non-habitat builders | 3.447 | 69.74 |
| 55-100 m | **C+DWOR - RB+SB+DWOR** | Megafauna; Benthic; Sessile; Suspension Feeders; Solitary; Non-habitat builders | 3.167 | 72.907 |
| 100-200 m | **C+CN+DWOR - SB** | Megafauna; Benthic; Sessile; Filter Feeders; Solitary; Non-habitat builders | 35.64 | 35.64 |
| 100-200 m | **C+CN+DWOR - SB** | Megafauna; Benthic; Sessile; Scavenger/Predator; Solitary; Non-habitat builders | 16.94 | 52.58 |
| 100-200 m | **C+CN+DWOR - SB** | Megafauna; Benthic; Sessile; Suspension Feeders; Colonial; Non-habitat builders | 13.2 | 65.78 |
| 100-200 m | **C+CN+DWOR - SB** | Megafauna; Benthic; Sessile; Suspension Feeders; Solitary; Habitat builders | 7.27 | 73.05 |
